# Synthesis, Structure, and Properties of CuBiSeCl_2_: A Chalcohalide Material with Low Thermal Conductivity

**DOI:** 10.1021/acs.chemmater.4c00188

**Published:** 2024-04-23

**Authors:** Cara J. Hawkins, Jon A. Newnham, Batoul Almoussawi, Nataliya L. Gulay, Samuel L. Goodwin, Marco Zanella, Troy D. Manning, Luke M. Daniels, Matthew S. Dyer, Tim D. Veal, John B. Claridge, Matthew J. Rosseinsky

**Affiliations:** † Department of Chemistry, Materials Innovation Factory, University of Liverpool, 51 Oxford Street, Liverpool L7 3NY, U.K.; ‡ Stephenson Institute for Renewable Energy and Department of Physics, University of Liverpool, Liverpool L69 7ZF, U.K.

## Abstract

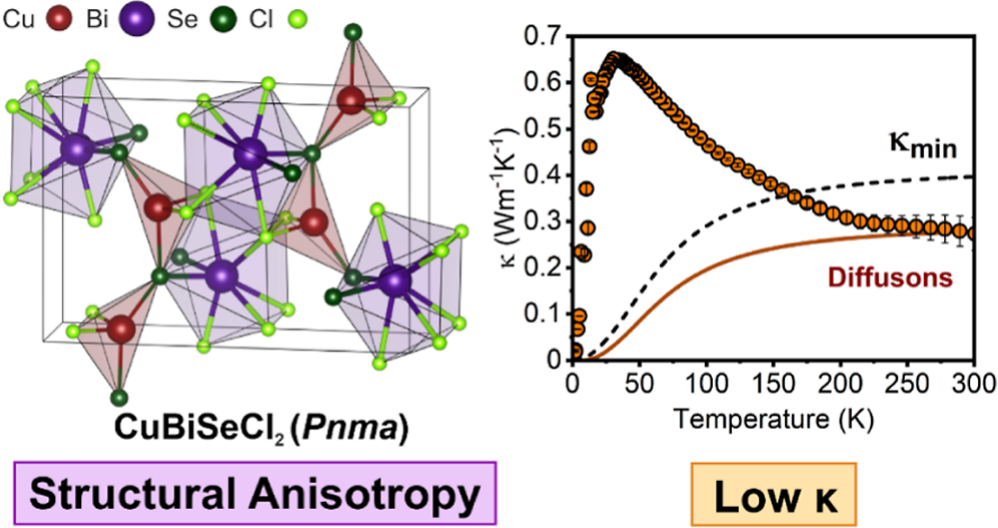

Mixed anion halide-chalcogenide materials have recently attracted attention for a variety of applications, owing to their desirable optoelectronic properties. We report the synthesis of a previously unreported mixed-metal chalcohalide material, CuBiSeCl_2_ (*Pnma*), accessed through a simple, low-temperature solid-state route. The physical structure is characterized through single-crystal X-ray diffraction and reveals significant Cu displacement within the CuSe_2_Cl_4_ octahedra. The electronic structure of CuBiSeCl_2_ is investigated computationally, which indicates highly anisotropic charge carrier effective masses, and by experimental verification using X-ray photoelectron spectroscopy, which reveals a valence band dominated by Cu orbitals. The band gap is measured to be 1.33(2) eV, a suitable value for solar absorption applications. The electronic and thermal properties, including resistivity, Seebeck coefficient, thermal conductivity, and heat capacity, are also measured, and it is found that CuBiSeCl_2_ exhibits a low room temperature thermal conductivity of 0.27(4) W K^–1^ m^–1^, realized through modifications to the phonon landscape through increased bonding anisotropy.

## 1 Introduction

Employing a multiple anion approach to material design provides a route to achieving potentially improved properties beyond single anion compositions.^1^ In comparison with single-anion materials, mixed-anion compounds are less well characterized. The myriad opportunities afforded through the exploration of novel multianionic phase spaces have recently stimulated more research into this area. Mixed anion chemistry drives material design in two ways: by increasing the number of degrees of freedom available in a system and by modifying the bonding within a material. This can lead to increased structural anisotropy, changes in dimensionality, and the evolution of novel structure types.^2,3^ Structural changes arising from such bonding variations facilitate the emergence of new physical phenomena^4−6^ and also provide opportunities for optoelectronic property tuning through modifications to the electronic structure.^7,8^ A multiple anion approach has already been successfully applied to engineer desirable properties in a variety of energy materials, including battery cathode materials,^9,10^ solid-state electrolytes,^11^ and thermoelectrics.^12−14^

Halide-chalcogenide, or “chalcohalide”, materials are an emerging class of mixed anion materials that contain one or more different metal cations, at least one chalcogenide anion, and at least one halide anion. The multifunctionality of these materials has made them a suitable platform for property tuning to suit a variety of applications, including photocatalysis^15^ and X-ray detection.^16^ In particular, they have gained attention as potential solar absorbers, with the aim of using a split anion approach^17^ to combine the desirable optoelectronic properties of lead halide perovskites (LHPs) with the enhanced stability of chalcogenide-based materials.^18,19^ The opportunities for wider compositional and structural exploration through employing the mixed anion chemistry of chalcohalide materials make them a valuable starting point for this task.^20^ Several mixed-metal chalcohalides are suitable for photovoltaic (PV) applications, including Sn_2_SbS_2_I_3_^21,22^ and CuBiSCl_2_. The latter was recently applied in a single-junction cell by Ming and co-workers and demonstrated exceptional environmental stability, a suitable band gap of 1.44 eV, and a PCE of 1%.^23^

Here, we report the synthesis, crystal structure, and thermal, electronic, and optical properties of CuBiSeCl_2_, a previously unreported mixed-metal chalcohalide material. CuBiSeCl_2_ is a p-type semiconductor with a low thermal conductivity of 0.27(4) W K^–1^ m^–1^ and a band gap of 1.33(2) eV. The material was also determined to be stable in ambient air for over a month.

## 2 Experimental Section

### 2.1 Reagents

CuCl (99.999%), Bi_2_Se_3_ (99.995%), and BiCl_3_ (99.995%) were purchased from Sigma-Aldrich and were used without further purification. Reagents were stored and handled in an Ar-filled glovebox with <0.1 ppm of O_2_ and <0.1 ppm of H_2_O.

### 2.2 Synthesis of CuBiSeCl_2_

Powder samples of CuBiSeCl_2_ on a 1 g scale were synthesized by grinding stoichiometric amounts of reagents in an agate mortar, pressing the reagent mix into an 8 mm diameter pellet, and loading into a flame-dried quartz ampule of dimensions 1 cm × 20 cm. The ampule was evacuated to 1 × 10^–3^ mbar and sealed under a vacuum. The sample was placed horizontally into a high-temperature oven. The sample was then heated using a ramp rate of 5 °C/min, fired at 430 °C for 10 h, and cooled to 200 °C using a ramp rate of 0.1 °C/min before shutting off the oven and allowing it to cool to room temperature. Single crystals of CuBiSeCl_2_ were grown using the same reaction conditions used for powder synthesis, but a constant cooling rate of 1 °C/h was used between 430 °C and room temperature.

### 2.3 Powder X-ray Diffraction

Preliminary phase identification was carried out using a Rigaku SmartLab diffractometer with Mo Kα radiation (λ = 0.7107 Å) in the Debye–Scherrer geometry. Samples were sealed inside 0.2 mm diameter borosilicate capillaries in an Ar-filled glovebox. 16 h scans were conducted on a Bruker D8 Advance with monochromatic Cu Kα_1_ radiation (λ = 1.54056 Å) in the Debye–Scherrer geometry to obtain high quality data for structural refinement. A LaB_6_ internal standard was ground into the CuBiSeCl_2_ mixture to extract accurate lattice parameters during refinement. 12 hour scans were conducted on an X’Pert Panalytical diffractometer with monochromatic Co Kα_1_ radiation (λ = 1.788965 Å) in the Bragg–Brentano geometry to quantify the extent of preferred orientation in pressed pellets of CuBiSeCl_2_. Rietveld refinements against CuBiSeCl_2_ powder x-ray diffraction (PXRD) data were conducted using TOPAS Academic V5.^24^ The background, lattice parameters, atomic positions, and atomic displacement parameters were refined against the data; a Chebyshev polynomial function was used to model the background; and the peak shapes were modeled using Stephens orthorhombic functions.^25^ Structural refinement parameters are available in the Supporting Information (S17).

### 2.4 Single Crystal X-ray Diffraction

A black single crystal with a platelet shape was selected under a polarizing microscope and then studied by single crystal x-ray diffraction (SCXRD) on beamline I19, Diamond Light Source, Didcot, U.K. using silicon double crystal monochromatic synchrotron radiation (λ = 0.6889 Å, Pilatus 2 M detector).^26^ The synchrotron data were collected with the sample at 100 K. Cell refinement and data reduction were performed using Xia^27^ and Dials programs.^28^ The structure was solved and refined using SHELX-2013,^29^ implemented through Olex2.^30^ The final residual factors converged to *R*1 = 0.0119 and *wR*2 = 0.0114 for reflections with *I* > 2σ (*I*). Single crystal solution structure refinement parameters, isotropic and anisotropic thermal displacement parameters, and main bond distances are available in the Supporting Information (S13–S16).

### 2.5 Compositional Analysis

Scanning electron microscopy (SEM) was performed on a Tescan S8000. Pelletized powder and single crystal samples were attached to an adhesive carbon tape stuck on an aluminum SEM stub. To reduce the charging effects, the samples were coated with a thin layer of carbon. Energy dispersive X-ray (EDX) spectroscopy and wavelength dispersive X-ray (WDX) spectroscopy were performed on the same instrument using X-MaxN and Wave detectors from Oxford Instruments. WDX calibrations for the different elements were obtained by measuring the WDX spectra of appropriate standards. Standard purity was confirmed by using X-ray diffraction and electron microscopy. Quantification was performed by using Aztec software.

### 2.6 Transmission Electron Microscopy Imaging

Transmission electron microscopy (TEM) images were collected on CuBiSeCl_2_ particles by using a JEOL JEM 2100+ transmission electron microscope, and compositions were confirmed by using EDX. Samples were loaded onto a Au TEM grid, which was then mounted to a JEOL common specimen holder (EM-21010) before being introduced into the microscope.

### 2.7 Spark Plasma Sintering

Dense pellets (∼88% theoretical density) were obtained by spark plasma sintering (SPS) of the phase-pure CuBiSeCl_2_ powder at 300 MPa and 270 °C for 5 min in a 10^–3^ mbar vacuum using a commercial Thermal Technology LLC DCS10 furnace. Powder samples (∼0.45 g) were pressed in a 10 mm diameter, graphite-foil-lined tungsten carbide die set (with 6% Co binder). Heating and pressure ramp rates were set to 20 °C/min and 100 MPa/min, respectively. The temperature was monitored through a borehole on the side of the die set using a thermocouple. After pressing, the pellets were lightly polished with SiC paper to remove the graphite foil from the pellet surface.

### 2.8 UV–Vis Spectrometry

Diffuse reflectance of CuBiSeCl_2_ powder was measured using an Agilent Cary 5000 instrument between 200 and 2500 nm with a step size of 1 nm. Calibration to 100 and 0% reflectance was performed prior to measurement using a PTFE standard and a light trap, respectively. The band gap was determined from a Tauc plot using a method described by Makuła et al.^31^

### 2.9 X-ray Photoelectron Spectroscopy

Core-level and valence band X-ray photoelectron spectroscopy (XPS) measurements were collected at HarwellXPS, Didcot, U.K. XPS analysis was performed using a Kratos Axis SUPRA XPS fitted with a monochromatic Al Kα X-ray source (*h*ν = 1486.6 eV), a spherical sector analyzer, and 3 multichannel resistive plates, 128 channel delay line detectors. All data were recorded at 150 W and a spot size of 700 × 300 μm^2^. Survey scans were recorded at a pass energy of 160 eV, and high-resolution scans were recorded at a pass energy of 20 eV. Electronic charge neutralization was achieved using a magnetic immersion lens. All sample data were recorded at a pressure below 10^–8^ Torr and temperature of 294 K. Data were analyzed using CasaXPS v2.3.19PR1.0. Peaks were fit with a Shirley background prior to component analysis. Mixed Gaussian–Lorentzian lineshapes [GL(50)] were used to fit components. All binding energies were measured with respect to the Fermi edge of a Ag foil reference sample.

The ionization potential (IP) of CuBiSeCl_2_ was determined by collecting the secondary electron cutoff (SEC) region using a monochromatic Al Kα SPECS X-ray source with a PSP Vacuum Technology hemispherical electron energy analyzer with a mean radius of 120 mm. Pass energies of 2 eV for the SEC, 10 eV for core levels, and 50 eV for survey scans were used to measure the emitted photoelectrons. Measurements were performed in an ultrahigh-vacuum chamber with a base pressure of 2 × 10^–10^ mbar. SEC data were recorded at 16 W to prevent overloading the analyzer. A 10 V bias was applied to the sample to remove any effects from the material work function. The Cu 2p region was also measured to use as a reference. The spectrometer resolution was determined to be 0.40 eV by fitting a Fermi–Dirac function convolved with a Gaussian function to the Fermi edge of the Ag foil.

### 2.10 Properties Measurement

SPS prepared pellets were cut into semicircles with 1.13 mm thickness and 4.9 mm radius by using a low-speed, diamond-blade saw for Seebeck coefficient, electronic resistivity, and thermal conductivity measurements. Copper electrodes were attached to the pellet using Ag epoxy and left to dry overnight. The offcuts from the pellets were used for powder diffraction and compositional analysis. The thermal conductivity, electronic conductivity, and Seebeck coefficient were measured simultaneously between 2 and 300 K using two probe geometry on a dense pellet of CuBiSeCl_2_ using the Thermal Transport Option (TTO) on a Quantum Design physical properties measurement system (PPMS). Heat capacity measurements were performed on a small fragment of dense pellet, with a mass of 0.0125 g, of CuBiSeCl_2_ using the Heat Capacity Option (HCO) on the PPMS. The sample was mounted using N grease. An addenda measurement was performed on the sample holder and grease prior to mounting and measuring the pellet fragment. Both the addenda and pellet fragment measurements were performed between 2 and 300 K.

### 2.11 Environmental Stability

The stability of CuBiSeCl_2_ in ambient air and water was determined as part of this study. The water stability of CuBiSeCl_2_ was determined by mixing CuBiSeCl_2_ powder with distilled water for 10 min and pipetting several drops of the suspension onto a glass slide. The slide was then left under ambient conditions for 24 h to allow the water to evaporate. PXRD data were measured before and after water exposure. The air stability was determined by sprinkling CuBiSeCl_2_ powder onto a glass slide, leaving the slide under ambient conditions for several weeks, and measuring PXRD data at regular time intervals.

### 2.12 Computational Details

Electronic structure calculations were performed using the periodic density functional theory (DFT) program, Vienna Ab initio simulation package (VASP).^32^ Core electrons were treated using the projector-augmented wave approach^33^ with Bi 5d electrons included as valence electrons. The hybrid functional, HSE06,^34^ was used with the inclusion of spin–orbit coupling (SOC) effects^35^ and a plane-wave cut off energy of 400 eV. The structure of CuBiSeCl_2_ was relaxed until forces fell below 0.01 eV Å^–1^ with a Γ-point centered 10 × 5 × 3 *k*-point grid. The high-symmetry path for calculation of the band structure was obtained using the automatic flow library.^36^

## 3 Results and Discussion

**Figure 1:**
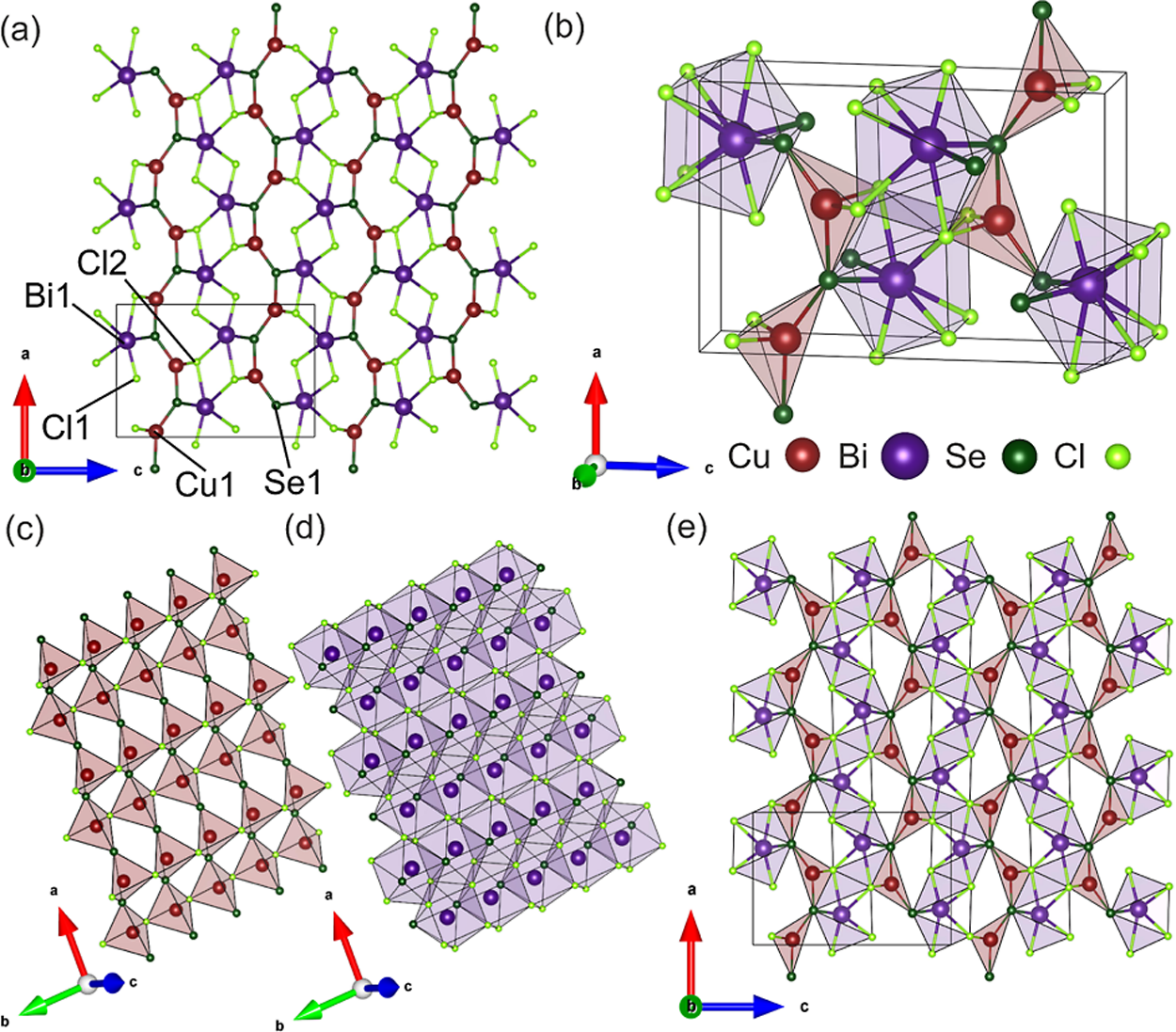
(a) Ball and stick model of the structure of CuBiSeCl_2_ (*Pnma*) as solved from SCXRD data viewed along the *b* axis and including the unit cell with labeled atomic sites. (b) Unit cell of CuBiSeCl_2_ depicting bonding environments of CuSe_2_Cl_2_ tetrahedra and BiSe_2_Cl_6_ polyhedra. (c) Layers of CuSe_2_Cl_2_ tetrahedra and (d) BiSe_2_Cl_6_ polyhedra, which are stacked alternately along the *c* direction in CuBiSeCl_2_. (e) Polyhedral structure of CuBiSeCl_2_ viewed along the *b* axis.

Figure 1a represents the crystal structure of CuBiSeCl_2_ solved from SCXRD data, viewed along the *b* axis. CuBiSeCl_2_ adopts the orthorhombic symmetry of *Pnma*, with lattice parameters *a* = 8.78415(6) Å; *b* = 3.99803(3) Å; and *c* = 13.13998(9) Å. In this structure, the oxidation states of the species are Cu^+^, Bi^3+^, Se^2–^, and Cl^–^, confirmed with the analysis of core-level binding energies from XPS (S1–S8). The unit cell of CuBiSeCl_2_ is depicted in Figure 1b and highlights the local bonding environments present in the overall structure. Cu^+^ exhibits a coordination number of four, with two Cu–Se bonds and two Cu–Cl bonds forming CuSe_2_Cl_2_ tetrahedra. Bi^3+^ is eight coordinate, forming two Bi–Se bonds and six Bi–Cl bonds to create square antiprismatic BiSe_2_Cl_6_ polyhedra. These copper- and bismuth-centered polyhedra are arranged such that two distinct layers are formed within the structure (Figure 1c,d). Layers of CuSe_2_Cl_2_ tetrahedra, which are connected over common Cl–Cl edges along the *b* direction and share a Se vertex in the *a* direction (Figure 1c), alternate with layers of Bi polyhedra, which face-share along the *b* direction and share two Cl–Cl edges in the *a* direction (Figure 1d). These layers are stacked vertically along the *c* direction such that a single CuSe_2_Cl_2_ tetrahedron is interconnected via edge sharing of two Cl–Se edges with two neighboring BiSe_2_Cl_6_ polyhedra, forming the overall CuBiSeCl_2_ structure depicted in Figure 1e.

Cu^+^ and Bi^3+^ are displaced from the center of their respective environments, visible in Figure 1e. Though it is tetrahedrally coordinated, Cu^+^ occupies an octahedral environment created by the Se^2–^ and Cl^–^ anions. Within this environment, Cu^+^ is displaced in the direction of the Cl–Cl octahedral edge to achieve sufficient valence. Bi is then displaced in the direction of the now under-bonded Cl^–^ at the octahedral corner. The origin of these displacements can be understood through comparisons with known compounds that exhibit similar structures: CuBiSCl_2_ (*Cmcm*) and MnBiSe_2_Br (*Pnma*).

**Figure 2:**
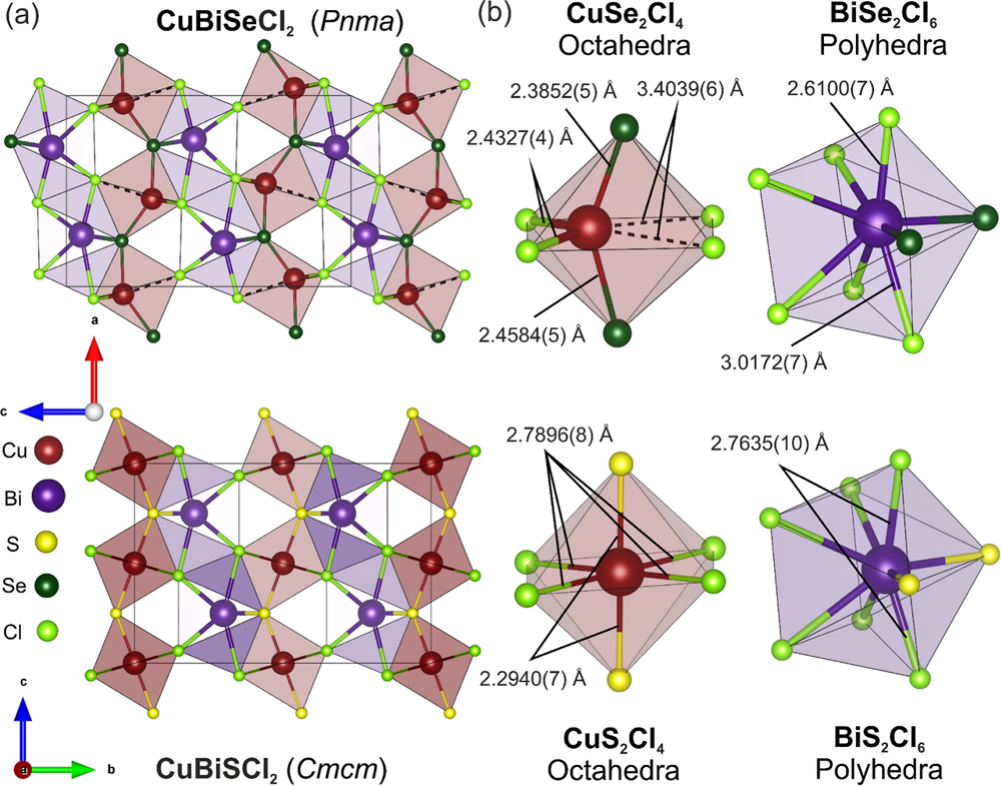
(a) Comparison of the unit cell of CuBiSeCl_2_ (*Pnma*) viewed along the *b* axis (top) with the unit cell of the higher symmetry CuBiSCl_2_ (*Cmcm*) viewed along the *a* axis (bottom). To highlight the structural differences between the two materials, Cu is shown in an octahedral environment in CuBiSeCl_2_, with the longer Cu–Cl bond represented by a dashed line to indicate that this bond is not physically observed. Furthermore, an additional row of CuSe_2_Cl_4_ octahedra are shown to allow for easier comparison of the CuBiSCl_2_ and CuBiSeCl_2_ unit cells. (b) Local bonding environments of CuChCl_4_ octahedra and BiCh_2_Cl_6_ polyhedra (Ch = S, Se) in CuBiSCl_2_ and CuBiSeCl_2_ are shown with specific Cu–Cl and Cu–Ch bonds labeled to highlight the variation in bond length with increasing anion size. Fully labeled CuChCl_4_ octahedra and BiCh_2_Cl_6_ polyhedra can be found in the Supporting Information (S9).

CuBiSCl_2_ adopts the higher symmetry *Cmcm* space group^40^ and exists as a “post-perovskite” structure, first observed in a high-pressure polymorph of MgSiO_3_.^41^ The structure of CuBiSCl_2_ is made up of alternating layers of CuS_2_Cl_4_ octahedra and BiS_2_Cl_6_ polyhedra and shows the same packing of polyhedra as the selenide compound, but the polyhedra are much less distorted in the sulfide analogue. Figure 2 presents a structural comparison of CuBiSeCl_2_ and CuBiSCl_2_. Here, octahedral Cu environments are included in the CuBiSeCl_2_ unit cell and local environments to allow for better comparison between the two materials, though the long Cu–Cl “bonds” [3.4039(6) Å] are shown with dashed lines to indicate they are not physically reasonable. The following discussion will also consider the octahedral CuSe_2_Cl_4_ environment, again for ease of comparison.

The comparison between the structures of CuBiSeCl_2_ and CuBiSCl_2_^37^ indicates that increasing the chalcogen anion size is the primary driving force for the transition to lower symmetry observed for CuBiSeCl_2_. A group–subgroup relation scheme detailing the symmetry lowering that occurs between CuBiSCl_2_ and CuBiSeCl_2_ is included in the Supporting Information (S10). Figure 2a depicts the unit cells of both structures and Figure 2b shows labeled local bonding environments of CuBiSeCl_2_ and CuBiSCl_2_ to highlight the variation in bond lengths between the two materials.

Figure 2b highlights the anisotropic bond lengths present in the CuSe_2_Cl_4_ octahedral environment, with the contracted Cu–Cl bonds in the direction of Cu^+^ displacement [2.4327(4) Å] almost 1 Å shorter than the longer Cu–Cl “bonds” opposite [3.4039(6) Å]. The Cu–Se bond lengths also show some anisotropy, though these differences are less pronounced than in the Cu–Cl bond lengths. As the Cu^+^ is displaced toward the Cl^–^ at the octahedral vertex, it is also displaced in the *a* direction toward one of the Se^2–^ species. Figure 2b depicts the shortening of the Cu–Se bond in the direction of displacement [2.3856(9) Å] and the lengthening of the other bond [2.4573(9) Å]. The Cu^+^ displacement only happens in the *a*–*c* plane, and there is no displacement along the *b* direction.

Bi^3+^ exhibits the same coordination of six chloride ions and two chalcogen ions in both materials; however, the Cu^+^ displacements in CuBiSeCl_2_ also affect Bi^3+^, which is displaced from the center of the polyhedra, introducing differences in the Bi–Cl and Bi–Se bond lengths. As shown in Figure 2b, the Bi1–Cl1 bond [2.6100(6) Å] contracts as Bi shifts toward the under-bonded Cl of the CuSe_2_Cl_4_ octahedra, which induces lengthening of the Bi1–Cl2 bond [3.0172(7) Å] opposite. Again, Bi^3+^ displacement happens only in the *a*–*c* plane.

More detail about the origin of Cu^+^ displacements in CuBiSeCl_2_ can be understood using bond valence sum (BVS) analysis (S12–S14). The location of Cu^+^ on the 4*a* site at the center of the octahedron in CuBiSeCl_2_ would yield a BVS of 0.716, indicating that Cu^+^ is underbonded in such an environment. Displacement from the center to the 4*c* position toward the Cl–Cl edge achieves higher valence for Cu^+^ of 0.913. For comparison, in the reported structure for CuBiSCl_2_, Cu^+^ occupies a site at the center of the octahedron with a BVS of 0.865. The volume of the CuSe_2_Cl_4_ environment increases from 23.8 Å^3^ in CuBiSCl_2_ to 33.1 Å^3^ in CuBiSeCl_2_, which is the reason the Cu^+^ displacement is observed in the latter. This is supported by calculation of BVS maps (Figures S13 and S14) that show a cuboidal region with a side length of ∼0.7 Å about the center of the octahedron where Cu^+^ would achieve a valence of 1. Calculation of the BVS parameters for octahedrally coordinated Cu^+^ positioned on the 4*c* site in the experimentally observed *Pnma* CuBiSeCl_2_ material confirms the tetrahedral coordination of Cu^+^ by showing that the long Cu–Cl “bond” [3.4039(6) Å] contributes minimally to the overall valence of Cu^+^.

We also note that in the structure of CuBiSCl_2_, a large *U*_iso_ of 0.068 Å^2^ is observed for Cu, which may infer some unresolved structural displacement around this position. This is further supported by BVS maps calculated for CuBiSCl_2_, which depict a cuboidal region of similar size to that observed for CuBiSeCl_2_.

Considering the coordination chemistry of Cu^+^ can also provide insights into the structural displacements present in CuBiSeCl_2_. Typically, Cu^+^ forms 3 and 4-fold coordinate environments. In compounds with similar chemistries to CuBiSeCl_2_ like Cu_3_BiS_3_ and CuIn_*x*_Ga_1–*x*_Se_2_, Cu^+^ preferentially occupies a tetrahedral coordination. In addition to this, Xiao et al. report that, as of yet, there are no reported compounds whereby Cu^+^ is 6-fold coordinated with halides.^38^

The crystal structure of CuBiSeCl_2_ is most similar to that of MnBiSe_2_Br, which also crystallizes in the space group *Pnma*.^39^ A structural comparison between the unit cells of CuBiSeCl_2_ and MnBiSe_2_Br can be found in the Supporting Information (S11). Again, though Cu^+^ is tetrahedrally coordinated in CuBiSeCl_2_, we will consider the CuSe_2_Cl_4_ octahedra for ease of comparison with the MnSe_4_Br_2_ octahedra in MnBiSe_2_Br.

The structure of MnBiSe_2_Br consists of layers of MnSe_4_Br_2_ octahedra and BiSe_5_Br_3_ polyhedra and, like the Cu displacement in CuBiSeCl_2_, MnBiSe_2_Br exhibits a deviation in the position of Mn from the center of the MnSe_4_Br_2_ octahedra and toward the Br–Br octahedral edge. Bi occupies split sites in MnBiSe_2_Br, with the Bi1 site exhibiting an occupancy of 0.97 and the Bi2 site possessing an occupancy of 0.03. In CuBiSeCl_2_, the Cu is displaced much more significantly from the center of the octahedra compared to the displacement of Mn observed in MnBiSe_2_Br. The differences in the cationic displacement magnitude observed in the two structures are partly due to the stoichiometric variation in the local environments, which results in different coordination and bonding.

In the MnSe_4_Br_2_ octahedra, the two Br^–^ ions occupy equatorial cis positions around the central Mn^2+^ ion with the Se^2–^ ions occupying the remaining four sites (S9). In contrast, in the CuSe_2_Cl_4_ octahedra the four Cl^–^ ions occupy all equatorial positions and the two Se^2–^ ions are positioned trans on the axial positions. One factor that influences the magnitude of the cationic displacement in CuBiSeCl_2_ relative to MnBiSe_2_Br is the size of the ionic radii in the octahedral environment. In the MnSe_4_Br_2_ environment, all of the anionic species exhibit similar ionic radii (Br 1.96 Å, Se 1.98 Å),^40^ which causes minimal displacement of Mn^2+^. The difference in anion size in the CuSe_2_Cl_4_ octahedra is more significant (Cl 1.81 Å, Se 1.98 Å) and results in a more anisotropic displacement of Cu^+^. The variation in structural displacements achieved in these materials by using different coordination environments in three similar structures suggests that using a mixed anion approach provides a route to establishing control over specific structural features.

**Figure 3:**
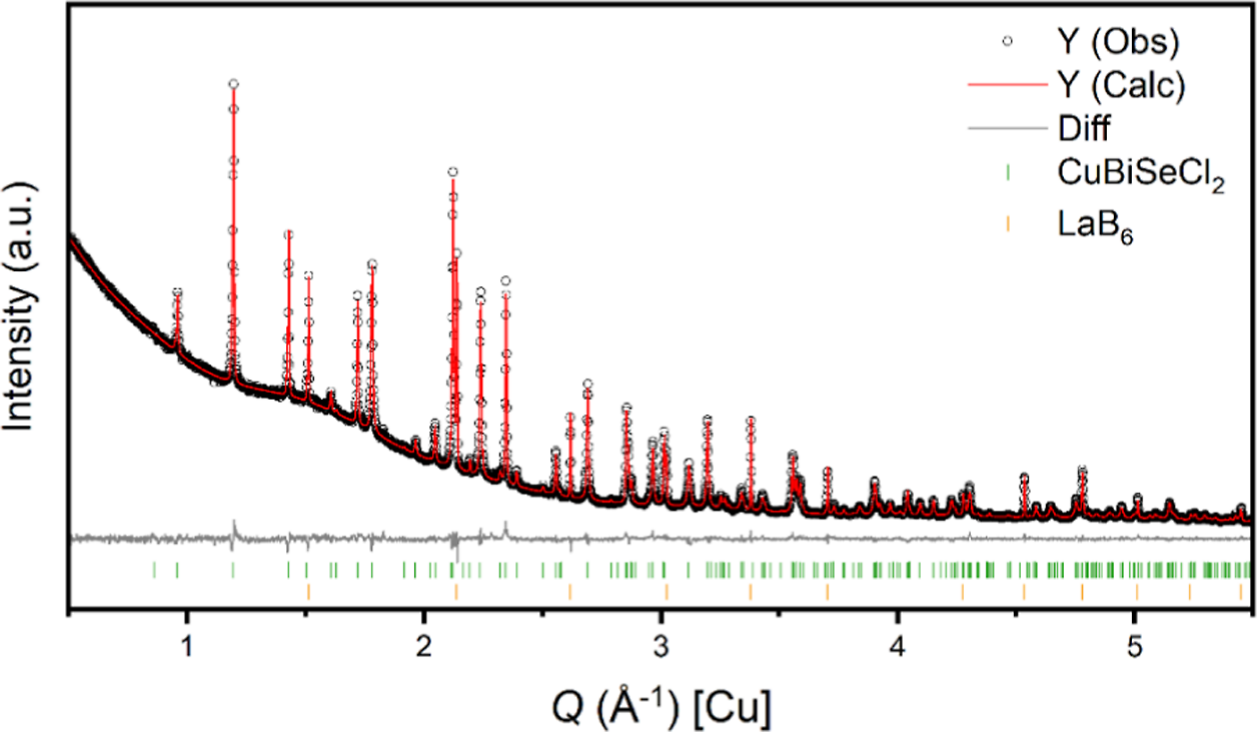
Rietveld analysis of the PXRD data measured for CuBiSeCl_2_ with LaB_6_ used as an internal standard.

Rietveld analysis of CuBiSeCl_2_ PXRD data, shown in Figure 3, was used to confirm the structural model determined from SCXRD data and demonstrate the phase purity of the bulk samples. TEM images (S21) show the particles to be needle-like structures of approximately 1.5 μm × 0.25 μm. Compositional analysis was performed on both single crystal and powder samples of CuBiSeCl_2_ to corroborate the stoichiometry determined from the single crystal model, as shown in S22–S26. SEM–EDX elemental mapping was performed on a single crystal of CuBiSeCl_2_ to confirm the homogeneous distribution of elements as well as the absence of oxygen in the structure. Single crystal EDX elemental mapping in Figure S22 indicates that Cu, Bi, Se, and Cl are all uniformly distributed in the sample and the presence of oxygen is minimal. To further confirm the material stoichiometry and account for the significant overlap of Bi^3+^ and Cl^–^ peaks in the EDX spectra, SEM–EDX and WDX were also measured on CuBiSeCl_2_ powder, and these data are depicted in S23 and S24. SEM–WDX was used to quantify the Cu/Bi and Bi/Cl ratios, while SEM–EDX measured the Bi/Se ratio. The overall composition of CuBiSeCl_2_ was determined to be Cu_1.10(3)_Bi_1.0(4)_Se_1.15(5)_Cl_2.17(3)_ consistent with the stoichiometry established from the structural solution.

**Figure 4:**
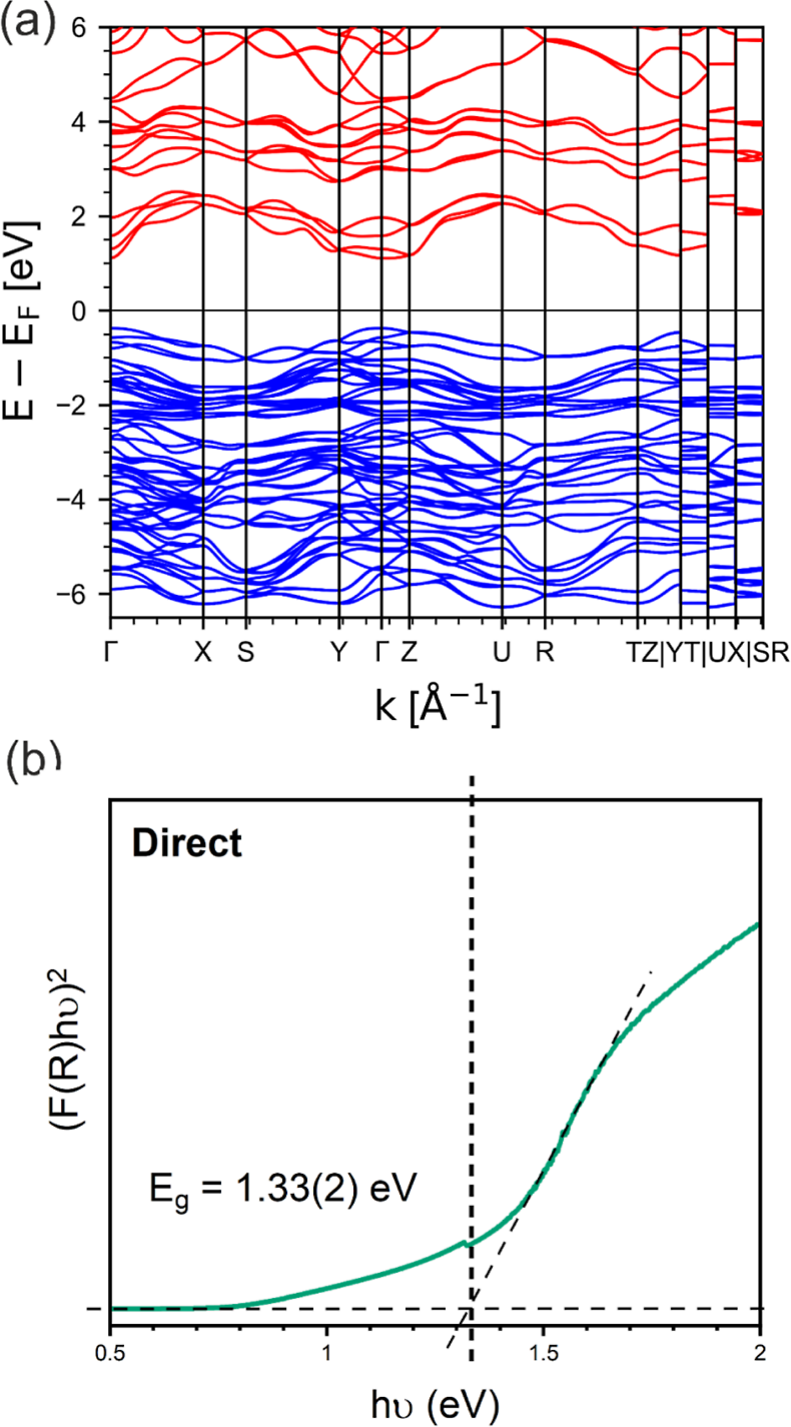
(a) Band structure of CuBiSeCl_2_ calculated using the HSE06 functional with SOC contributions. Valence band electronic states are shown in blue and the conduction band electronic states in red. (b) Tauc plot derived from UV–vis spectrometry measurements. The direct band gap for CuBiSeCl_2_ is extrapolated using the Tauc plot and determined to be 1.33(2) eV.

The band structure and effective masses were calculated for CuBiSeCl_2_ to provide insights into the optical band gap and mobility of charge carriers within the material as well as elucidate possible applications. The band structure of CuBiSeCl_2_ is shown in Figure 4a.

The direct band gap calculated from this band structure is found to be 1.5 eV, suggesting that CuBiSeCl_2_ may have potential as a solar absorber. Interestingly, the calculated direct and indirect bandgaps are very close in energy such that the band gap in CuBiSeCl_2_ can be considered to be quasi-direct. This occurs since the conduction band in the Γ → *Z* direction is extremely flat close to the Γ point and dips slightly as it goes away from the Γ point such that the computed conduction band minimum (CBM) is actually displaced slightly away from the Γ point itself. This leads to an indirect gap that is only very slightly smaller than the direct gap (0.1 meV). This effect is also observed in the band structure of CuBiSCl_2_, whereby, when SOC is accounted for, the CBM shifts from Y toward Γ, but this energy difference is very small (10 meV); hence, the band gap in this material is considered to be quasi-direct.^23^

To provide insight into the effect of the physical structure on the electronic transport properties in CuBiSeCl_2_, the band structures of the reported sulfide analogue CuBiSCl_2_ (*Cmcm*) and the hypothetical, higher-symmetry CuBiSeCl_2_ (*Cmcm*) material were also calculated (S28). The associated charge carrier effective masses are reported for the experimentally observed CuBiSeCl_2_ (*Pnma*), the hypothetical higher symmetry CuBiSeCl_2_ structure (*Cmcm*) and CuBiSCl_2_ (*Cmcm*) in Table 1. The effective masses for CuBiSeCl_2_ are reported for the Γ → *X*, Γ → *Y*, and Γ → *Z* directions. For the *Cmcm* structures, the effective masses are reported for the *Y* → *X*_1_, *Y* → Γ, and *Y* → *T* reciprocal space paths.

**Table 1:** Electron (*m*_e_*) and Hole (*m*_h_*) Effective Masses (Relative to the Mass of an Electron, m_0_) Calculated as Part of This Study from the Band Structures of CuBiSCl_2_ (*Cmcm*), CuBiSeCl_2_ (*Cmcm*), and CuBiSeCl_2_ (*Pnma*) in the *k*_*x*_, *k*_*y*_, and *k*_*z*_ Directions

CuBiSeCl_2_ (*Pnma*)^a^	*k*_*y*_ (Γ → *Y*)	k_*z*_ (Γ → *Z*)	k_*x*_ (Γ → *X*)
*m*_e_* (m_0_)	1.0	12	0.2
*m*_h_* (m_0_)	8	1.6	4

CuBiSeCl_2_ (*Cmcm*)	*k*_*x*_ (*Y* → *X*1)	*k*_*y*_ (*Y* → Γ)	*k*_*z*_ (*Y* → T)
*m*_e_* (m_0_)	3	4.2	0.7
*m*_h_* (m_0_)	2.5	0.7	1.3
CuBiSCl_2_ (*Cmcm*)			
*m*_e_* (m_0_)	0.25	3	0.65
*m*_h_* (m_0_)	2.9	0.75	1.1

a Indicates that the effective masses for the *Pnma* structure of CuBiSeCl_2_ have been reordered relative to the *Cmcm* structures such that the directions are related by *k*_*x*_ → *k*_*z*_, *k*_*z*_ → *k*_*y*_, and *k*_*y*_ → *k*_*x*_ to account for symmetry lowering effects between *Cmcm* and *Pnma* and allow for ease of comparison between different symmetries.

Though the band structure of CuBiSeCl_2_ exhibits visibly low dispersion, the calculated effective masses within CuBiSeCl_2_ are highly anisotropic, with particularly heavy electron effective masses in the *k*_*z*_ direction but much lighter electrons in the *k*_*x*_ direction.

The very light electron effective masses in the *k*_*x*_ direction result from the highly connected face-sharing Bi^3+^ polyhedra and corner-sharing Cu^+^ octahedra, creating “channels” for the charge carriers. These channels are visible in Figure 1a,d. In a similar way, the moderate effective masses in the *k*_*y*_ direction result from the edge-sharing Cu^+^ octahedra and face-sharing Bi^3+^ polyhedra, providing pathways for conductivity. Electrons are more mobile than holes in both the *k*_*x*_ and *k*_*y*_ directions.

The heavy electrons in the *k*_*z*_ direction may be a result of the displacement of Cu^+^ away from the octahedral center, with the reduced orbital overlap between Cu^+^ and Cl^–^ weakening the pathway for charge carriers and therefore reducing the conductivity through the network. Therefore, these anisotropic effective masses are likely to result from the anisotropic bonding in CuBiSeCl_2_ that arises due to the inclusion of mixed anions in the structure.

The calculated effective masses for all compounds are anisotropic, suggesting that this is characteristic of the structure of these materials. CuBiSCl_2_ (*Cmcm*) exhibits light effective masses along *k*_*x*_ (0.25 m_0_) and *k*_*z*_ (0.65 m_0_), with electrons being more mobile than holes in both directions. Substituting S for Se into the same *Cmcm* structure has the effect of increasing the electron effective masses observed along *k*_*x*_ (3 m_0_), with holes now being more mobile than electrons in this direction. The electron effective mass in the *k*_*z*_ direction also increases slightly (0.7 m_0_). Increases in the calculated effective masses observed going from the sulfide to the selenide *Cmcm* structures probably arise due to longer bond distances, which reduce the orbital overlap between atomic species.

Interestingly, when comparing with the calculated effective masses for the experimentally observed *Pnma* structure of CuBiSeCl_2_, it is found that m_e_ in the *k*_*z*_ direction of CuBiSCl_2_ decreases to 0.2 m_0_ in the corresponding *k*_*x*_ direction in *Pnma* CuBiSeCl_2_. Likewise, *m*_e_ along *k*_*x*_ also decreases from 3 to 1 m_0_ in the corresponding *k*_*y*_ direction between the *Cmcm* and *Pnma* structures of CuBiSeCl_2_, respectively.

The reduction in electron effective masses in the *k*_*x*_ and *k*_*z*_ directions between hypothetical CuBiSeCl_2_ (*Cmcm*) and experimentally observed CuBiSeCl_2_ (*Pnma*) corresponds to the Cu^+^ displacement, which occurs in the *a*–*c* plane. In particular, the electron effective mass in the *c* direction is substantially reduced as a result of the significant Cu^+^ displacement toward the octahedral edge in this direction, which affords increased orbital overlap within the structure. Consequently, the Cu^+^ displacement also has the effect of increasing m_e_ in the *k*_*y*_ direction in CuBiSeCl_2_ (*Pnma*) to 12 m_0_, which is a sharp increase compared to the analogous direction in CuBiSCl_2_ (*Cmcm*) (3 m_0_).

Though CuBiSeCl_2_ possesses heavy charge carriers in the *k*_*z*_ direction, this should not be a barrier to its implementation as a thin film for optical absorbing applications, provided it is grown in the correct orientation. For example, Sb_2_Se_3_ is a well-established solar cell absorber that also exhibits highly anisotropic effective masses.^41^ These anisotropic properties are overcome by orienting crystal growth perpendicular to the substrate, allowing for efficient transport along one axis.^42^ Saparov et al. also report a method to deposit preferentially oriented thin films,^43^ and depositing *a* axis-aligned CuBiSeCl_2_ could reduce the effects of the heavy charge carriers along the *c* axis on the material conductivity.

CuBiSeCl_2_ may be particularly suited to the deposition of orientated thin films as a result of the effects of the preferred orientation. Analysis of PXRD data measured on CuBiSeCl_2_ powder and pellets in reflection geometry reveals that CuBiSeCl_2_ particles preferentially orient in the [*h*00] and [00*l*] directions (S24) such that the [0*k*0] direction is oriented parallel to the surface normal direction, supported by refining the March–Dollase parameters using the Rietveld method.

Diffuse reflectance UV–vis spectrometry was measured on CuBiSeCl_2_ powder to ascertain the optical band gap. The band gap was determined from a Tauc plot, as shown in Figure 4b, and is found to be 1.33(2) eV, in good agreement with the value derived from the band structure calculations (1.5 eV). Complete agreement between the experiment and calculations is not expected since the DFT calculates the band structure at 0 K, whereas UV–vis diffuse reflectance was measured at 300 K. However, as the temperature is increased, the calculated band gap will decrease, indicating even better agreement between calculation and experiment. CuBiSeCl_2_ is measured to have a lower band gap than CuBiSCl_2_, with the value increasing from 1.33(2) to 1.44 eV between the two compounds, respectively. A reduction in band gap with increasing Se content is observed in materials with related chemistries, including CuSb(S_*x*_Se_1–*x*_)_2_,^44^ BiCuS_*x*_Se_1–*x*_O,^45^ and BaCu_2_Sn(Se_*x*_S_1–*x*_)_4_.^46^

A long sub-bandgap absorption (Urbach) edge is present in the Tauc plot of CuBiSeCl_2_, indicative of either structural (static) or thermal (dynamic) disorder present in the material.^47^ More specifically, the size of this Urbach edge is directly influenced by lattice vibrations in the material,^48^ and in LHPs, it has been shown that increased electron–phonon scattering enlarges the Urbach edge.^49^ Modeling of heat capacity data from CuBiSeCl_2_ (see Section 3.2) confirms the presence of several localized phonon modes, suggesting that a complex landscape of lattice vibrations in the material influence band edge disorder, resulting in the extended Urbach edge.

**Figure 5:**
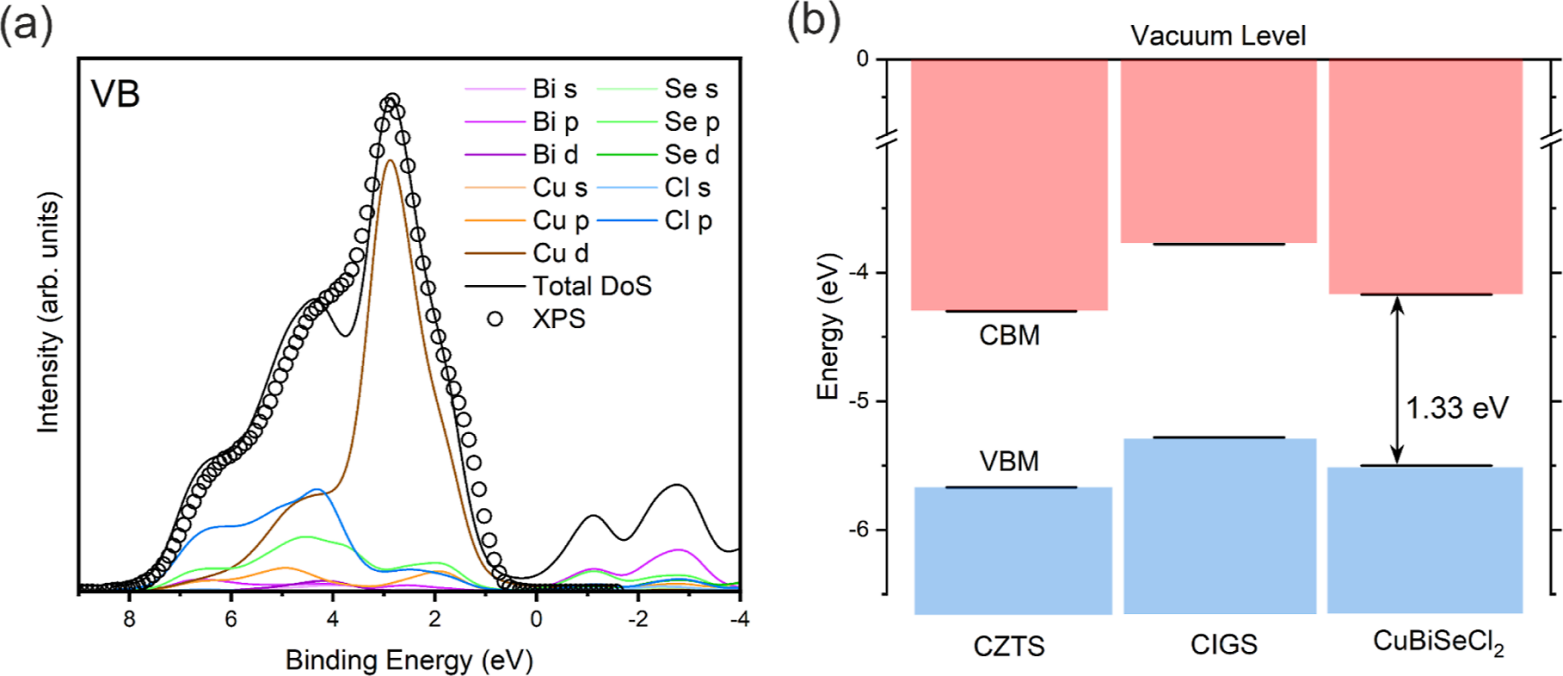
(a) CuBiSeCl_2_ valence band DoS, calculated using the HSE06 functional with additional SOC contributions and broadened to account for realistic photoemission processes, plotted with valence band XPS data experimentally measured on CuBiSeCl_2_ powder. (b) Band alignments of CuBiSeCl_2_ in comparison with optical absorbers with similar chemistries, CZTS and CIGS. The conduction band and valence band states are colored red and blue, respectively.

To understand the electronic structure of CuBiSeCl_2_ in more detail, experimental measurement of the valence band using XPS was combined with partial density of states (pDoS) calculations. Figure 5a shows valence band XPS compared to pDoS that has been corrected to account for realistic photoemission processes, including lifetime broadening and spectrometer resolution. The broadening processes used are discussed in greater detail in the Supporting Information (S29 and S30).

The corrected pDoS indicates that Cu 3d states dominate the top of the valence band, while Cl 3p states occupy higher binding energies much deeper into the valence band. Se 4p, Cu 3p, and, to a lesser extent, Bi 5p/5d states cover a broad range of binding energies between 0 and 6 eV in the valence band. There is some hybridization between the Cu 3d, Se 4p, and Cl 3p states at higher binding energy in the valence band, likely due to the overlap of Cu, Se, and Cl orbitals in the tetrahedral CuSe_2_Cl_2_ bonding environment. Similarly, hybridization between Bi 5p and Se 4p/Cl 3p states at the bottom of the conduction band may arise from the Bi–Se/Bi–Cl bonding in the Bi polyhedra.

A sharp peak arising from Cu orbital contributions is also observed in the pDoS of similar chalcogenide and chalcohalide materials, including Cu_3_BiS_3_ (*P*2_1_2_1_2_1_)^50^ and Bi_2_CuSe_3_I (*C*2/*m*),^51^ in which Cu occupies different bonding environments. This suggests that Cu has a commanding effect on both chalcogen and halogen orbitals across different structures with similar chemistries, heavily influencing the top of the valence band. Comparisons between the pDoS of CuBiSCl_2_ and CuBiSeCl_2_ reveal that the valence band consists of a hybridization of Cu 3d, Ch 3p (where Ch = S, Se), and Cl 4p states in both materials, though the number of Se 3p states in the CuBiSeCl_2_ valence band is lower than the number of S 3p states in the CuBiSCl_2_ valence band. This is attributed to the reduced orbital overlap in CuBiSeCl_2_ as a result of longer bond distances.

Excellent agreement is observed between the XPS data and pDoS, suggesting that the DFT calculations provide accurate insights into the electronic character of the material. To enable the development of thin films and prototype devices based on CuBiSeCl_2_, it is essential to understand the band alignments of the material to allow the selection of suitable device structures and n-type window layers. Figure 5b depicts the band alignments for CuBiSeCl_2_ alongside well-established semiconductors with similar chemistries, Cu_2_ZnSnS_4_ (CZTS)^52^ and CuIn_*x*_Ga_1–*x*_Se_2_ (CIGS).^53^ By collecting the SEC region using very low-power XPS, it was possible to determine the IP and the valence band minimum (full description in the Supporting Information S32–S34). The CBM could then be located using the band gap, determined from diffuse reflectance UV–vis Spectroscopy, and found to be 1.33(2) eV, in good agreement with computational predictions. CdS has already been demonstrated as a successful window layer for CZTS and CIGS^54,55^ and, given the comparable band alignments, could also find application as a suitable window layer for devices based on CuBiSeCl_2_.

The environmental stability of CuBiSeCl_2_ was characterized to understand the powder degradation over time with exposure to air and moisture. CuBiSeCl_2_ powder was found to be stable in ambient air for over 1 month (S35). Analysis of PXRD data measured at regular time intervals shows that there is no change in the data taken after 10 min and 3 weeks of air exposure. However, after 9 weeks in ambient conditions, broad peaks consistent with the formation of BiOCl become visible. After mixing with water, depositing onto a glass slide, and drying for 24 h, it was observed that the black powder underwent a slight color change to pink around the very edges. A comparison of PXRD data from before and after water exposure reveals the appearance of small impurity peaks, consistent with the formation of BiOCl (S36).

### 3.1 Electronic Properties

**Figure 6:**
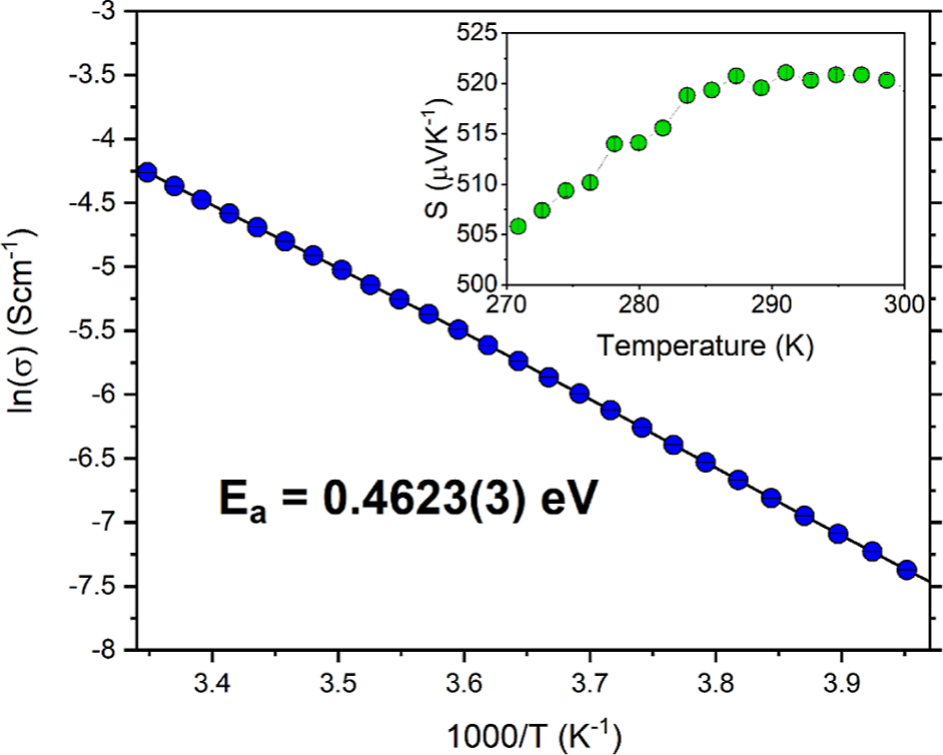
Arrhenius plot calculated from measuring the resistivity of CuBiSeCl_2_ parallel to the pressing direction between 250 and 300 K, with Seebeck coefficient measurement between 270 and 300 K inset. The activation energy is determined to be 0.4623(3) eV.

The electronic resistivity and Seebeck coefficient were measured on a dense pellet (∼88%) of CuBiSeCl_2_, parallel to the pressing direction, between 2 and 300 K (Figure 6) to understand the electronic transport within the material. The pellet was highly textured, as described above, with a preferential orientation in both the *a* and *c* directions (S24). Below 250 K, the resistance of CuBiSeCl_2_ became too high for any meaningful resistivity or Seebeck coefficient data to be collected; hence, these properties are only shown at approximately 250 K.

At 300 K, CuBiSeCl_2_ is measured to have a large, positive Seebeck coefficient of 519.0(6) μVK^–1^, confirming that the material is a p-type semiconductor.

The electronic resistivity of CuBiSeCl_2_ exhibits behavior typical of a semiconductor, and a large resistivity of ∼6376(3) Ωm is measured at 300 K, consistent with the prediction of heavy charge carriers. Using the Arrhenius equation and by plotting ln(σ), where σ is the electronic conductivity, as a function of 1000/*T*, the activation energy (*E*_a_) could be extracted from the gradient and was found to be 0.4623(3) eV.

For an intrinsic semiconductor, it is expected that *E*_a_ is half the band gap, which in this case would be equal to 0.65 eV. We attribute the lower observed *E*_a_ to the p-type conductivity of CuBiSeCl_2_, which results in an additional extrinsic charge carrier density. It is likely that the measured *E*_a_ corresponds to the ionization energy of an acceptor level positioned above the valence band, an effect that has been observed in other p-type semiconductors. For example, p-type CuAlO_2_ possesses a direct band gap of 3.47 eV and a 0.7 eV activation energy.^56^ Similarly, Ga_2_O_3_ exhibits an ultrawide 4.8 eV band gap, which is almost four times the *E*_a_ (1.24 eV).^57^ In both cases, the discrepancy between the band gap and activation energy is associated with the presence of an acceptor level above the valence band.

### 3.2 Thermal Properties

**Figure 7:**
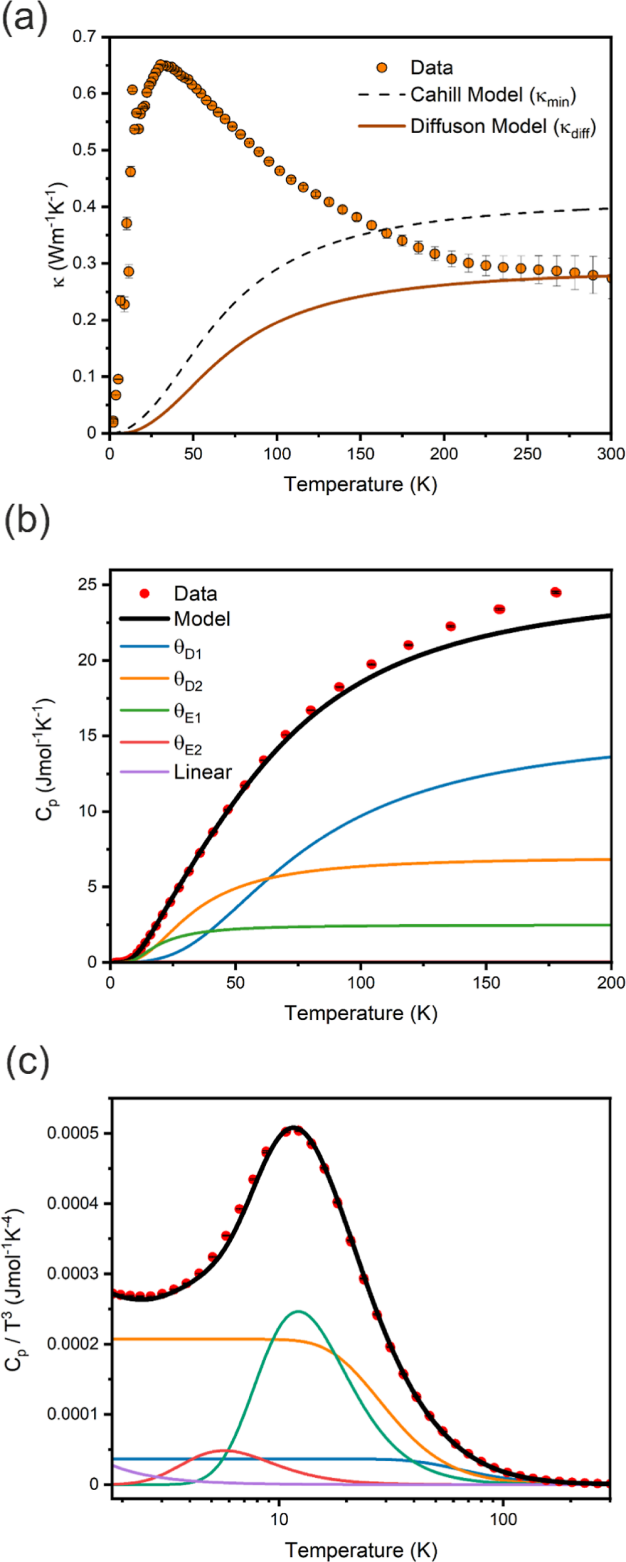
(a) Thermal conductivity of CuBiSeCl_2_ measured parallel to the pressing direction shown with Cahill (κ_min_) and diffuson-based (κ_diff_) models; fitted heat capacity data of CuBiSeCl_2_ shown as (b) *C*_*p*_ and (c) *C*_*p*_/*T*^3^ with Debye, Einstein, and linear components shown.

Along with the resistivity and Seebeck coefficient, the thermal conductivity was simultaneously measured on the same dense pellet of CuBiSeCl_2_, parallel to the pressing direction, between 2 and 300 K (Figure 7a). The behavior of the thermal conductivity of CuBiSeCl_2_ between 2 and 300 K is consistent with that of a crystalline material in which heat is transported by propagating phonons. At low temperatures, the thermal conductivity increases rapidly and peaks at 0.646(1) W K^–1^ m^–1^ at ∼35 K before decreasing to 0.27(4) W K^–1^ m^–1^ at 225 K, where it plateaus and is almost temperature-independent up to 300 K. Observation of the peak at ∼35 K in κ confirms the phonon-crystal characteristics of CuBiSeCl_2_.

The heat capacity of CuBiSeCl_2_ was measured on a fragment of the same dense pellet to provide insights into the behavior of phonons within the material and the origin of the particularly low thermal conductivity (Figure 7b,c). The data were modeled using a combination of linear, Debye, and Einstein terms, the parameters of which are summarized in Table 2.

**Table 2:** Heat Capacity Parameters Determined from Modeling Heat Capacity Measurements for CuBiSeCl_2_

compound	θ_D1_ (K)	θ_D2_ (K)	θ_E1_ (K)	θ_E2_ (K)	γ (J mol^–1^ K^–2^)
CuBiSeCl_2_	320	138	60	28	0.00009

The observation of an excess of specific heat at low temperatures, which often arises from highly localized vibrations,^58^ is accurately modeled using 2 Einstein temperatures, in addition to a linear term. This indicates the presence of several highly localized phonon modes within the structure.^59^ The Debye temperatures obtained from the heat capacity modeling enable the associated speeds of sound in CuBiSeCl_2_ to be calculated, which can provide information on the low thermal conductivity in the material. Further detail on the modeling used in this study may be found in Supplementary Information S37.

Using these values, the minimum thermal conductivity under the assumptions of the Cahill model (κ_min_) for CuBiSeCl_2_ was calculated (shown by the dashed line in Figure 7a).^60^ Unusually, the calculated minimum thermal conductivity (0.40 W K^–1^ m^–1^) is greater than the measured data (0.27 (4) W K^–1^ m^–1^) at 300 K. However, a diffuson model (κ_diff_) of the thermal conductivity, which includes the experimentally determined θ_D1_, θ_D2_, θ_E1_, θ_E2_, and *v*_s_, accurately captures the high temperature behavior of the measured data (shown in Figure 7a in red).^61^ This indicates that the thermal conductivity of the material must likely be understood through a two-channel model, where propagons dominate at low temperatures (resulting in the peak at 35 K), while diffusons dominate at higher temperatures (resulting in the plateau in thermal conductivity above 225 K).^62^ This effect has previously been observed in Ag_9_GaSe_6_ and was attributed to small interband spacings between phonon modes.^63^

The origin of the low thermal conductivity in CuBiSeCl_2_ can be understood by considering the structure and bonding present in the material. Achieving bonding anisotropy and structural anharmonicity are key design criteria for novel materials with ultralow thermal conductivities,^64,65^ as reducing or disrupting the bonding between species reduces the speed of sound and, therefore, the transport of heat through a material. The need for multiple Debye terms to accurately model the observed heat capacity can arise from the contributions of species with distinct bonds or masses within the structure. The higher-frequency represented by θ_D1_ (320 K) is associated with the more ionic character of Cu–Se/Cl bonds and the lower mass of Cu, while the lower frequency contributions of θ_D2_ (138 K) arise from the increased mass and more covalent bonding of Bi and can be understood when considering the local CuSe_2_Cl_2_ and BiSe_2_Cl_6_ environments within CuBiSeCl_2_. Certainly, the room temperature phonon mean free paths calculated from these Debye temperatures are of the order of 2–3 Å (S38 and S39), comparable to the interatomic distances within CuBiSeCl_2_, which indicates strong suppression of phonon propagation throughout the structure. In addition, 2 Einstein temperatures of θ_E1_ = 60 K and θ_E2_ = 28 K are required to model the excess specific heat evident from the experimentally measured heat capacity, the contributions of which represent those of localized oscillators with singular vibrational frequencies and thus nonpropagating modes. Again, the heavier mass of Bi is associated with a lower frequency θ_E2_ term. The higher-frequency Einstein temperature of θ_E1_ can be attributed to the local environment of Cu. As described above, Cu displaces from the center of an octahedral environment, observed in CuBiSCl_2_, to yield a tetrahedral CuSe_2_Cl_2_ environment in CuBiSeCl_2_. We note that the isotropic displacement parameter *U*_iso_ for the Cu site [0.01027(6) Å^2^] in the single crystal structure of CuBiSeCl_2_ solved at 100 K is approximately double that of any other species in the structure (Table S16). This difference in *U*_iso_ values for each site is significantly increased at room temperature from refinement of the model against PXRD data (Table S20), where the *U*_iso_ for Cu is 0.044(2) Å^2^, and the next largest is that of the Cl2 site [*U*_iso_ = 0.014(3) Å^2^]. Large *U*_iso_ values can be indicative of weak restoring forces and consequently weak bonding and anharmonicity.^66,67^ This indicates that Cu is loosely bound in the structure of CuBiSeCl_2_, despite the displacement to achieve higher bond valence. It is well established that loosely bound atoms can disrupt the propagating vibrational modes within the surrounding structural framework and are commonplace in materials such as clathrates and skutterudites;^68−71^ hence, Cu acts as a localized oscillator attributed to θ_E1_ and significantly enhances phonon scattering within the structure of CuBiSeCl_2_. The combination of multiple anions, and therefore bonding environments in CuBiSeCl_2_, infers a complex phonon landscape with localized vibrations that result in low speeds of sound and a low κ that is comparable to many other inorganic materials that exhibit low thermal conductivity.

For example, a similar effect is observed in the structurally complex Cu_4_Bi_4_Ch_9_ (Ch = S, Se) materials, whereby substitution of S for Se increases the volume of the coordination environment and reduces the localization of the Cu atoms.^72^ This increases the lattice anharmonicity in the selenide material, increasing the frequency of phonon scattering events, which manifests itself as a reduction in room temperature κ between Cu_4_Bi_4_S_9_ (0.44 K^–1^ m^–1^) and Cu_4_Bi_4_Se_9_ (0.29 W K^–1^ m^–1^). The similarity in κ between CuBiSeCl_2_ and Cu_4_Bi_4_Se_9_ points to the fact that increased Cu delocalization and the associated phonon scattering are contributing factors to the low thermal conductivity of CuBiSeCl_2_.

The thermal conductivity of CuBiSeCl_2_ is also comparable with that observed in other mixed anion materials, including 2D layered materials such as Bi_4_O_4_SeCl_2_. Gibson et al. employed the effect of bond anisotropy at the interfaces between layers of BiOCl and Bi_2_O_2_Se to achieve a material with ultralow thermal conductivity (0.1 W K^–1^ m^–1^) by minimizing the contribution of longitudinal and transverse phonons to heat transport.^64^ Bi_4_O_4_Cu_1.7_Se_2.7_Cl_0.3_, a system based on the intergrowth of Bi_2_O_2_Se and BiCuSeO layers, also realizes a low thermal conductivity [0.4(1) W K^–1^ m^–1^] through additional structural complexity.^2^

CuBiSeCl_2_ is an example of how anionic substitution can introduce structural anharmonicity, modifying the phonon landscape to drive the reduction of heat transport through the material.

## 4 Conclusions

We have reported the synthesis, crystal structure, and properties of CuBiSeCl_2_, a previously unreported p-type chalcohalide semiconductor with low thermal conductivity and a suitable band gap for solar-absorbing applications. Cationic displacements that arise from heterogeneous bonding induced by the different anions result in anisotropic charge carrier transport, with light effective masses observed in the *a* direction. As such, for the development of this material as a thin film for application as a potential solar absorber, the *a* axis-oriented deposition is suggested to utilize the superior effective masses along this direction. The low thermal conductivity observed in CuBiSeCl_2_ demonstrates how the combination of multiple anions can lead to separate structural motifs that significantly reduce κ, as evidenced through experimental heat capacity and structural refinement through loosely bound Cu species and bonding anharmonicity. As such, CuBiSeCl_2_ is an example of the opportunities for property modification through the application of mixed anion chemistry, as shown by the variations in the structural, electronic, and physical properties of CuBiSeCl_2_ relative to known materials with similar structures.

## Data Availability

The data underlying this study are openly available in University of Liverpool Data Repository at https://doi.org/10.17638/datacat.liverpool.ac.uk/2550.

## Supporting Information Available

Crystal structure details and comparisons; compositional analysis; TEM imaging; computational details and band structures; fitted core level XPS spectra; XPS survey spectra; environmental stability PXRDs; and description of thermal property modeling (PDF)
Crystallographic data for CuBiSeCl_2_ (CIF)

## Supplementary Material

cm4c00188_si_001.pdf

cm4c00188_si_002.cif
