# Correction to “Synthesis, Structure, and Properties of CuBiSeCl_2_: A Chalcohalide Material with Low Thermal Conductivity”

**DOI:** 10.1021/acs.chemmater.6c00606

**Published:** 2026-04-10

**Authors:** Cara J. Hawkins, Jon A. Newnham, Batoul Almoussawi, Nataliya L. Gulay, Samuel L. Goodwin, Marco Zanella, Troy D. Manning, Luke M. Daniels, Matthew S. Dyer, Tim D. Veal, John B. Claridge, Matthew J. Rosseinsky

The determination of the position of the ionization potential (IP) from the Valence Band Maximum (VBM) in Figure S34 in the Supporting Information should read “5.05 eV”, not “5.5 eV” as in the original publication. The band alignments for CuBiSeCl_2_ in Figure 5(b) must also be modified to reflect this correction.

Also in the Supporting Information, in S12, Table (ii), the Bond Valence Sums (BVS) calculated for Cu–Cl (1) and Cu–Cl (2) in the high symmetry CuBiSeCl_2_ structure should read “0.05692”, not “0.005692”.

The complete corrected Supporting Information and Figure 5 are presented herein. The authors note that these corrections do not change the primary conclusions of the paper.

**Figure 5:**
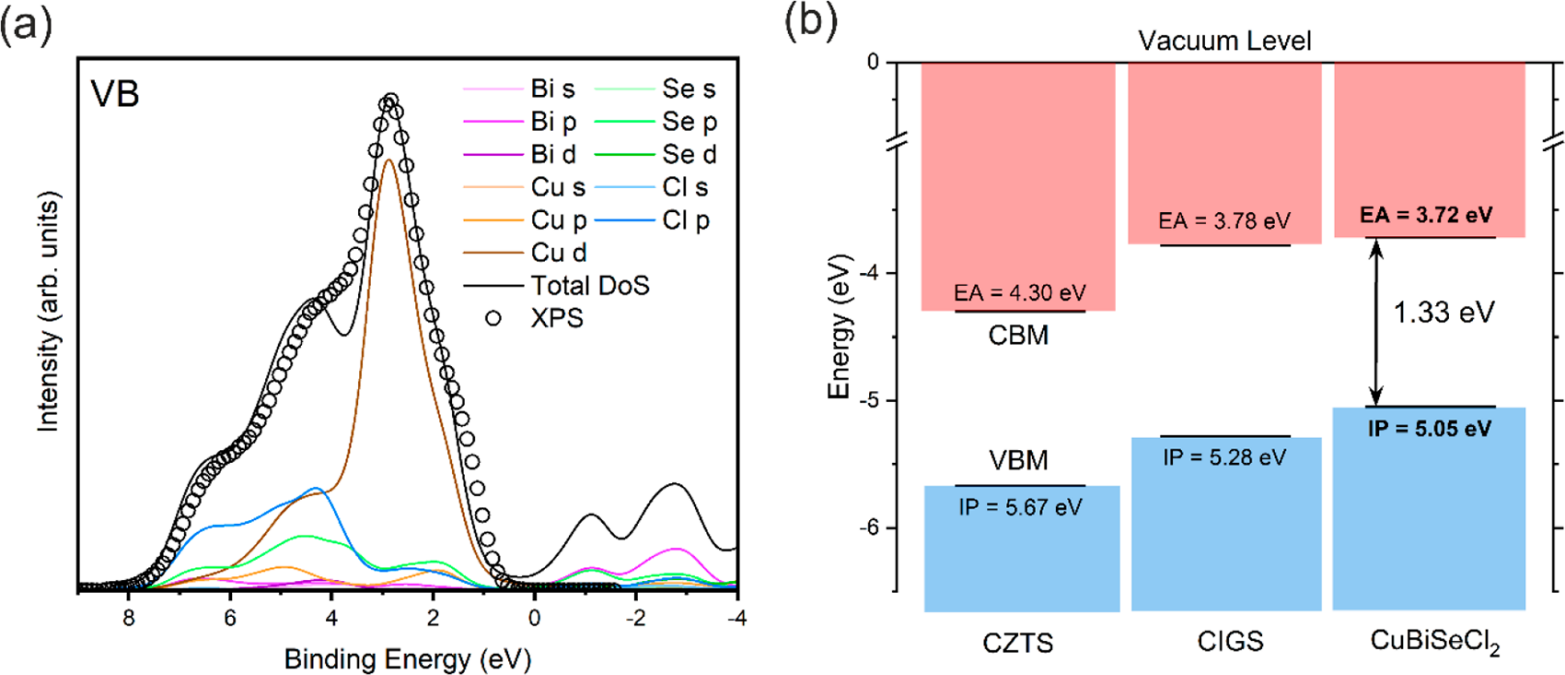
(a) CuBiSeCl_2_ valence band DoS, calculated using the HSE06 functional with additional SOC contributions and broadened to account for realistic photoemission processes, plotted with valence band XPS data experimentally measured on CuBiSeCl_2_ powder. (b) Band alignments of CuBiSeCl_2_ in comparison with optical absorbers with similar chemistries, CZTS and CIGS. The conduction band and valence band states are shown in red and blue, respectively.

## Supplementary Material



## Notes

Crystal structure details and comparisons; compositional analysis; TEM imaging; computational details and band structures; fitted core level XPS spectra; XPS survey spectra; environmental stability PXRDs; and description of thermal property modeling (corrected) (PDF)

