# Supplementary material for: Synthesis, Structure, and Properties of CuBiSeCl2: A Chalcohalide Material with Low Thermal Conductivity
Source: Chem Mater. 2024 Apr 23;36(9):4530–41. doi: 10.1021/acs.chemmater.4c00188 (PMC11099918; doi:10.1021/acs.chemmater.4c00188)
Supplement: Supplementary file 1 — cm4c00188_si_001.pdf [file cm4c00188_si_001.pdf]

Supplementary Information

Synthesis, Structure & Properties of $CuBiSeCl_2$: A Chalcohalide Material with Low Thermal Conductivity

Cara J. Hawkins[a], Jon A. Newnham[a], Batoul Almoussawi[a], Nataliya L. Gulay[a], Samuel L. Goodwin[a], Marco Zanella[a], Troy D. Manning[a], Luke M. Daniels[a], Matthew S. Dyer[a], Tim D. Veal[b], John B. Claridge[a] and Matthew J. Rosseinsky[a*]

a. Department of Chemistry, Materials Innovation Factory, University of Liverpool, 51 Oxford Street, Liverpool, L7 3NY, U.K.

b. Stephenson Institute for Renewable Energy and Department of Physics, University of Liverpool, Liverpool L69 7ZF, U.K.

*Email: m.j.rosseinsky@liverpool.ac.uk

The data underlying this study are openly available in University of Liverpool Data Repository at: https://datacat.liverpool.ac.uk/id/eprint/2550

The Supplementary Information is divided into the following sections

1. **Crystal Structure**
    i. **Oxidation State Determination**
    ii. **Structural Description**
2. **Compositional Analysis**
3. **Computational Details**
4. **Electronic Structure**
5. **Environmental Stability**
6. **Thermal Properties**

Crystal Structure

I. Oxidation State Determination

XPS was used to confirm the oxidation states of all species in $CuBiSeCl_2$ through comparison with literature reference values. Where possible, Full Width at Half Maximum (FWHM) and energy uncertainty values have been included from literature.

S1. XPS of Bi 4f core levels

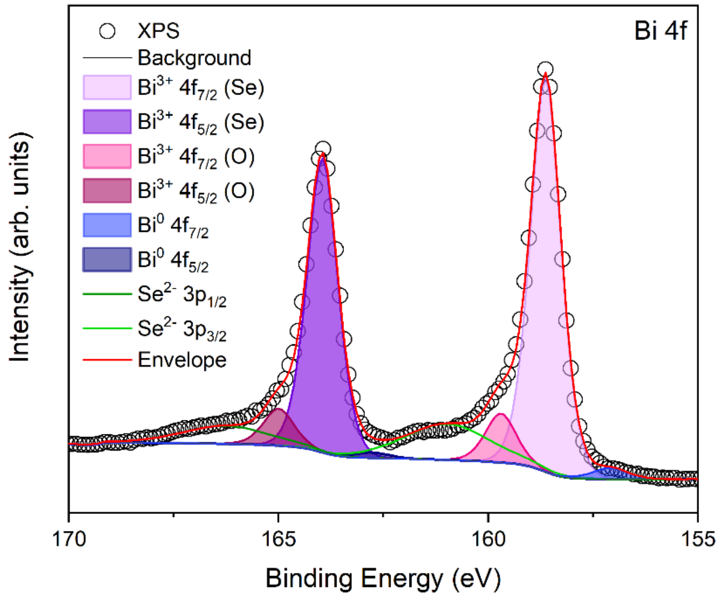

A small amount of oxygen contamination is visible in the Bi 4f core level spectra, attributed to surface oxidation. Note the overlap of the Bi 4f spectra with the Se 3p spectra, shown in green.

S2. Table of Bi 4f Binding Energies / Fitting Parameters

Bi 4f	Experimental		Literature	
Orbital	Binding Energy (eV)	FWHM (eV)	Binding Energy (eV)	FWHM (eV)
Bi^{3+} $4f_{7/2}$ (Se)	158.63	0.86	158.20 [1]	/
Bi^{3+} $4f_{5/2}$ (Se)	163.94	0.86	163.40 [1]	/
Bi^{3+} $4f_{7/2}$ (O)	159.72	0.86	160.00 [2]	/
Bi^{3+} $4f_{5/2}$ (O)	164.92	0.86	165.28 [2]	/
Bi^{0} $4f_{7/2}$	157.10	0.97	156.92 [2]	/
Bi^{0} $4f_{5/2}$	162.78	0.97	162.30 [1]	/

Se^{2-} $3p_{3/2}$	161.12	2.40	160.70 [3]	/
Se^{2-} $3p_{1/2}$	166.25	2.40	166.20 [4]	/

S3. XPS of Cu 2p core levels

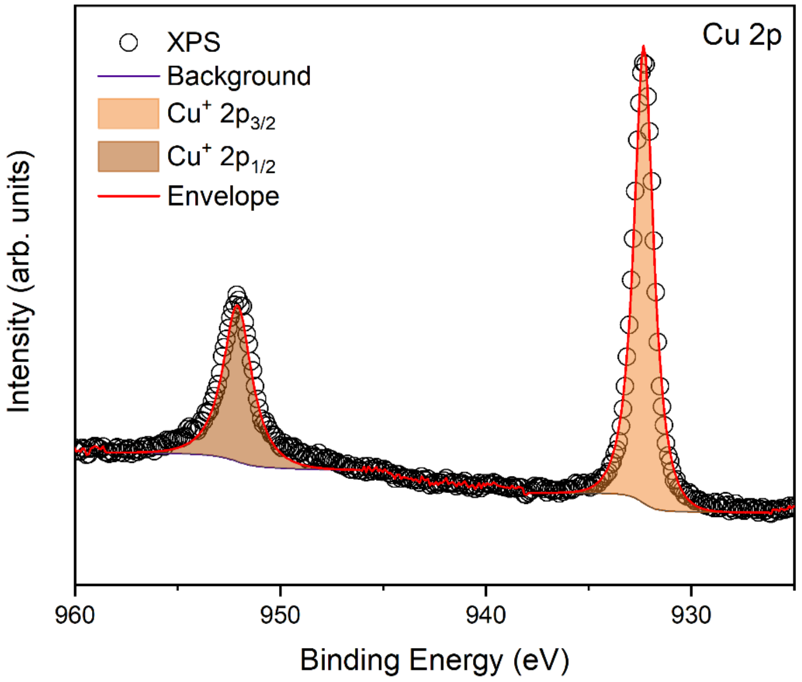

S4. Table of Cu 2p Binding Energies / Fitting Parameters

Cu 2p	Experimental		Literature	
**Orbital**	**Binding Energy (eV)**	**FWHM (eV)**	**Binding Energy (eV)**	**FWHM (eV)**
Cu^{+} $2p_{3/2}$	932.32	1.18	932.49 [5]	0.99
Cu^{+} $2p_{1/2}$	952.10	1.88	952.31 [5]	1.41

S5. XPS of Se 3d core levels

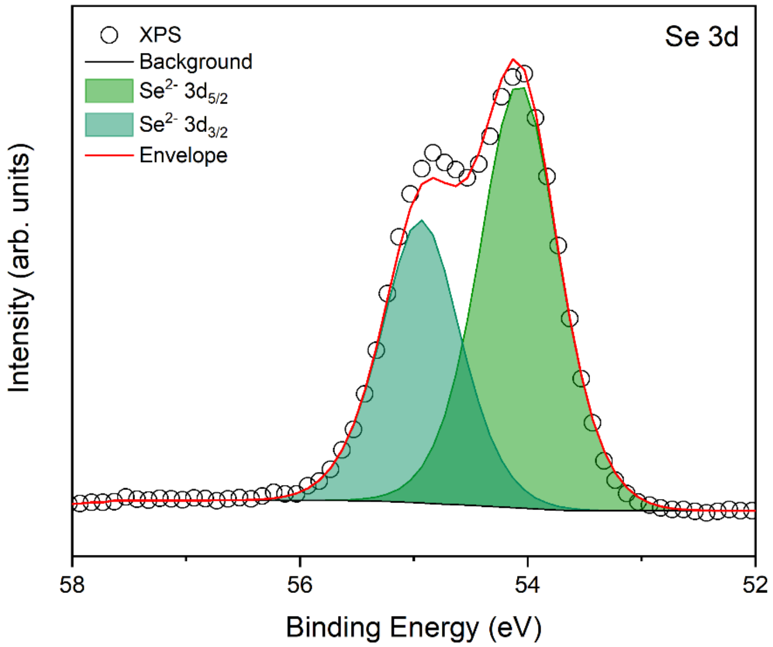

S6. Table of Se 3d Binding Energies / Fitting Parameters

**Se 3d**	**Experimental**		**Literature**	
**Orbital**	**Binding Energy (eV)**	**FWHM (eV)**	**Binding Energy (eV)**	**FWHM (eV)**
Se^{2-} $3d_{5/2}$	54.08	0.82	54.30 [5]	0.67
Se^{2-} $3d_{3/2}$	54.93	0.82	55.15 [5]	0.70

S7. XPS of Cl 2s / Cl 2p core levels

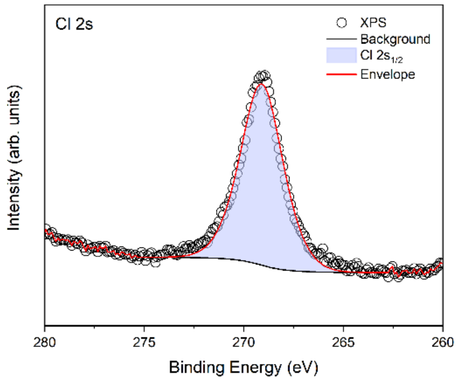

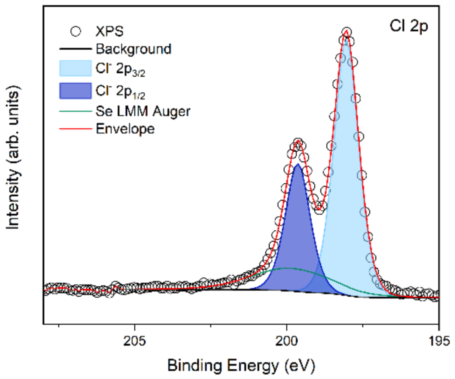

S8. Table of Cl 2s / 2p Binding Energies / Fitting Parameters

	Experimental		Literature	
**Orbital**	**Binding Energy (eV)**	**FWHM (eV)**	**Binding Energy (eV)**	**FWHM (eV)**
**Cl 2s**				
Cl^- $2s_{1/2}$	269.10	2.51	270 [6]	/
**Cl 2p**				
Cl^- $2p_{3/2}$	198.04	0.94	198.40 [7]	/
Cl^- $2p_{1/2}$	199.64	0.94	200.80 [8]	1.0
Se LMM Auger	199.58	3.40	/	/

Crystal Structure

II – Structural Description

S9. Structural comparison between local environments of $CuBiSCl_2$ (*Cmcm*)[9], $MnBiSe_2Br$ (*Pnma*)[10] and $CuBiSeCl_2$ (*Pnma*) unit cells, with fully labelled bond lengths. Note that in the $BiSe_5Br_3$ polyhedra, only the Bi-Se bond lengths for the 0.970 occupied Bi site are shown.

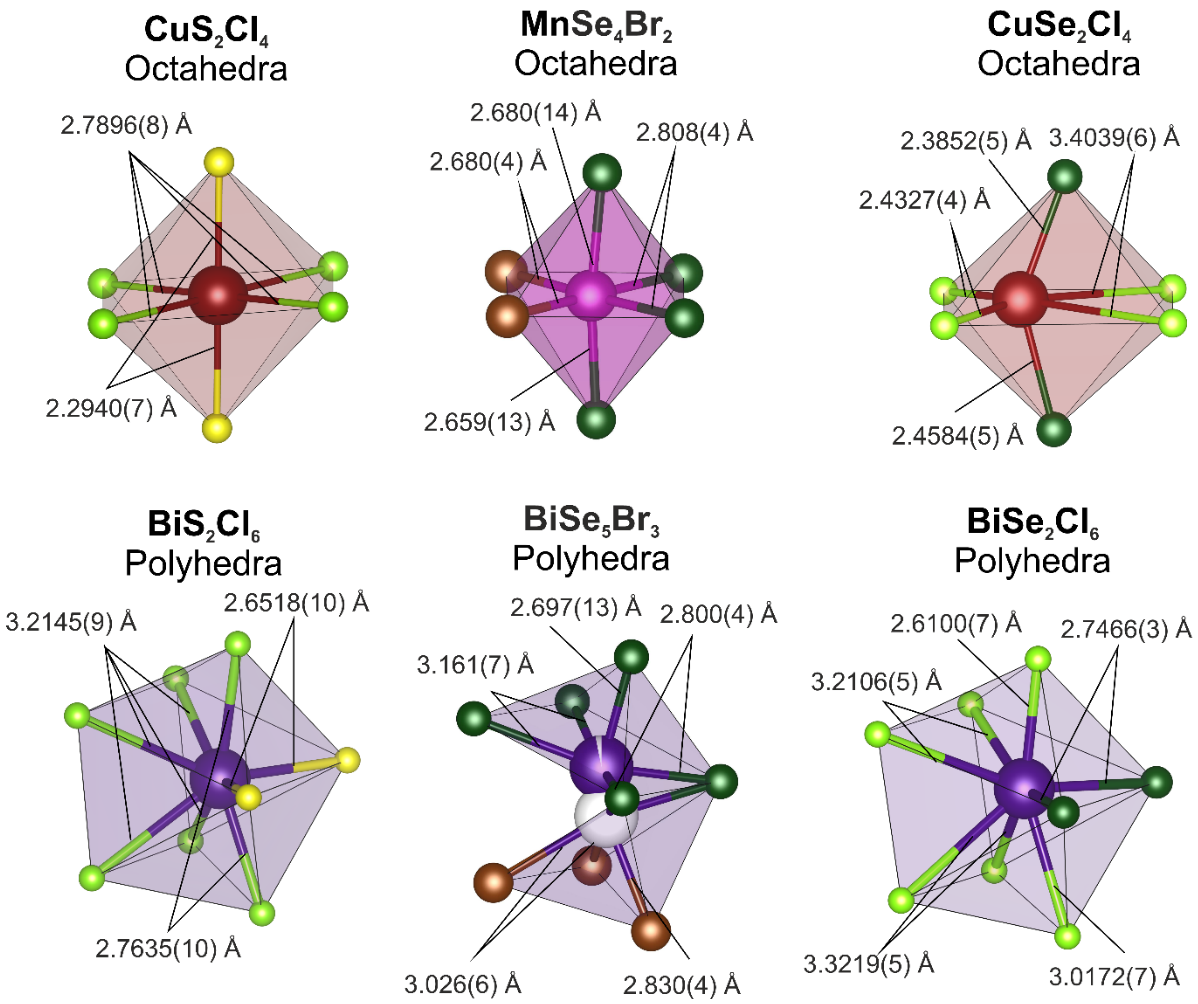

S10. Group-subgroup scheme in the Bärnighausen formalism[11-14] for the ($CuBiSCl_2$) type [6] subcell and the superstructure $CuBiSeCl_2$. The indices for the klassengleiche (k) symmetry reduction, the unit cell transformation, as well as the evolution of the atomic parameters are given. The atomic positions listed in S16 and S20 are transformed from the standard setting to match the theoretical model.

Cmcm
$CuBiSCl_2$

Cu: 4*a* 2/*m*..	Bi: 4*c* *m*2*m*	S: 4*c* *m*2*m*	Cl: 8*f* *m*...
0	0	0	0
0	0.7003	0.0627	0.3524
0	1/4	1/4	0.0618

↓ *k*2 *c*, *a*, *b*

Pnma
theoretical

Cu: 4*c* .*m*.	Bi: 4*c* .*m*.	S: 4*c* .*m*.	Cl1: 4*c* .*m*.	Cl2: 4*c* .*m*.
0.0000	0.2500	0.2500	0.0618	0.4383
3/4	3/4	3/4	3/4	3/4
0.7500	0.4503	0.8127	0.1024	0.1024

Pnma
$CuBiSeCl_2$
experimental

Cu: 4*c* .*m*.	Bi: 4*c* .*m*.	Se: 4*c* .*m*.	Cl1: 4*c* .*m*.	Cl2: 4*c* .*m*.
0.9596	0.2285	0.2394	0.0658	0.4408
3/4	3/4	3/4	3/4	3/4
0.8004	0.4475	0.8057	0.0955	0.1016

S11. Structural comparison between unit cells of $CuBiSeCl_2$ (*Pnma*) and $MnBiSe_2Br$ (*Pnma*)[10] Cu^+ is shown as octahedrally coordinated to allow for easier comparison between unit cells.

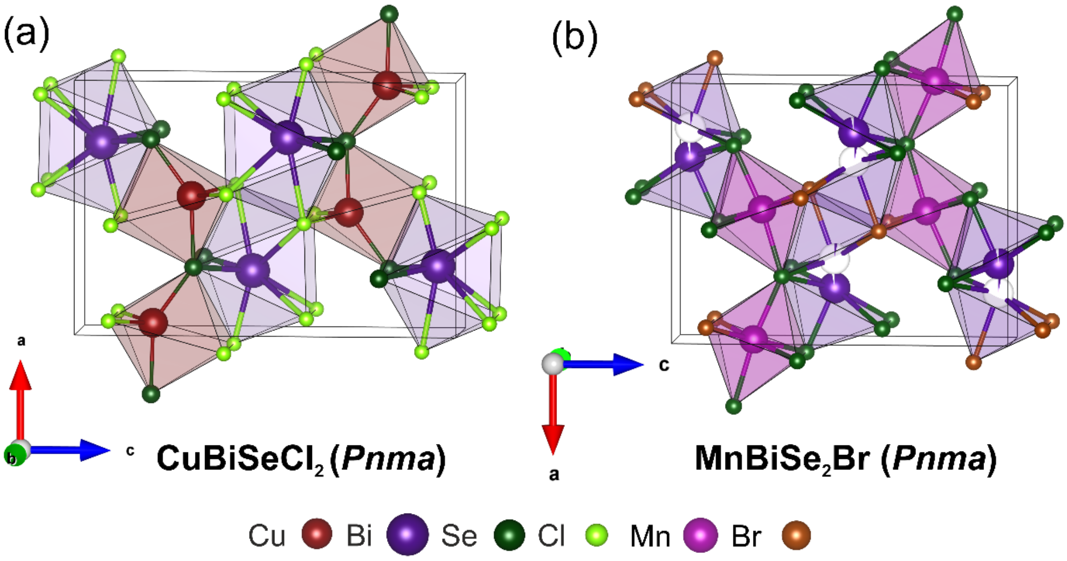

S12. Bond Valence Sums (BVS) calculated using bond valence parameters from [15] for (i) octahedrally and tetrahedrally coordinated Cu^+ positioned on the 4*c* site in the experimentally observed *Pnma* $CuBiSeCl_2$ material; (ii) octahedrally coordinated Cu^+ positioned on the 4*a* site in the hypothetical higher symmetry *Cmcm* $CuBiSeCl_2$ structure; (iii) octahedrally coordinated Cu^+ positioned on the 4*a* site in the experimentally observed *Cmcm* $CuBiSCl_2$ structure.

(i) Cu BVS (*Pnma*, experimentally observed $CuBiSeCl_2$).

**$CuBiSeCl_2$ (*Pnma*)**	**Length (Å)**	**v_i**
Octahedral Cu (4c)		
**Cu-Cl (Long)**	3.4035	0.0153
**Cu-Cl (Short)**	2.4334	0.2112
**Cu-Se (1)**	2.4573	0.2217
**Cu-Se (2)**	2.3856	0.2692
	**V_{Oct}**	**0.9439**
Tetrahedral Cu (4c)		
**Cu-Cl (Short)**	2.4334	0.2112
**Cu-Se (1)**	2.4573	0.2217
**Cu-Se (2)**	2.3856	0.2692
	**V_{Tet}**	**0.9132**

(ii) Cu BVS (*Cmcm*, theoretical, high symmetry $CuBiSeCl_2$).

**$CuBiSeCl_2$ (*Cmcm*)**	**Length (Å)**	**v_i**
Octahedral Cu (4a)		
**Cu-Cl (1)**	2.91845	0.005692
**Cu-Cl (2)**	2.91845	0.005692
**Cu-Se (1)**	2.42145	0.2443
**Cu-Se (2)**	2.42145	0.2443
	**V_{Oct}**	**0.7163**

(iii) Cu BVS (*Cmcm*, experimentally observed $CuBiSCl_2$).

**$CuBiSCl_2$ (*Cmcm*)**	**Length (Å)**	**v_i**
Octahedral Cu (4a)		
**Cu-Cl (1)**	2.7896	0.08063
**Cu-Cl (2)**	2.7896	0.08063
**Cu-Se (1)**	2.294	0.2711
**Cu-Se (2)**	2.294	0.2711
	**V_{Oct}**	**0.8647**

S13. Bond Valence Maps for (a) $CuBiSeCl_2$ (*Pnma*) and (b) $CuBiSCl_2$ (*Cmcm*). Bond valence sum maps were generated using the parameters published in [16]

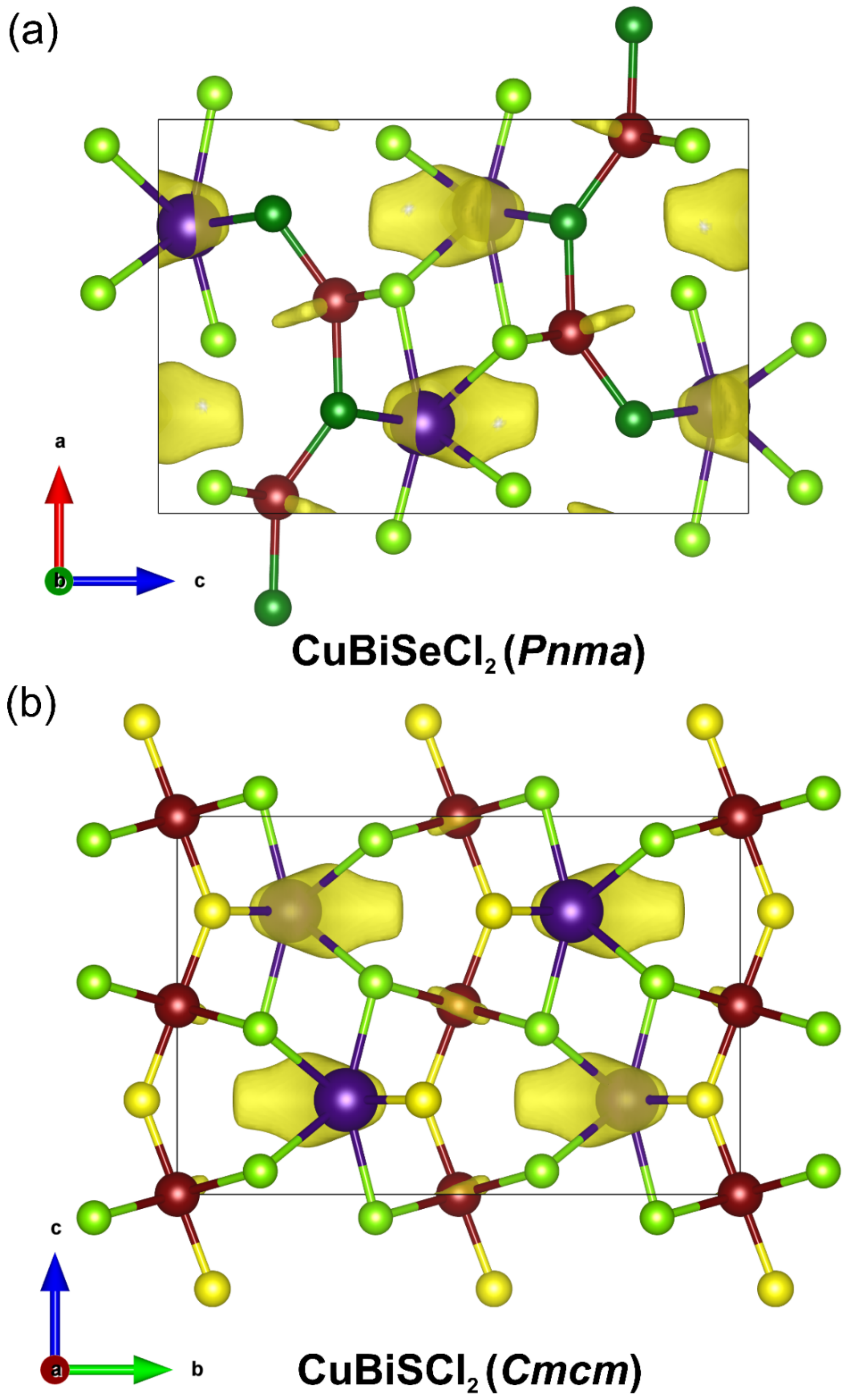

S14. Bond Valence Maps for (a) $CuSe_2Cl_4$ environments in $CuBiSeCl_2$ (*Pnma*) and (b) CuS_2Cl_4 environments in $CuBiSCl_2$ (*Cmcm*).

(i)

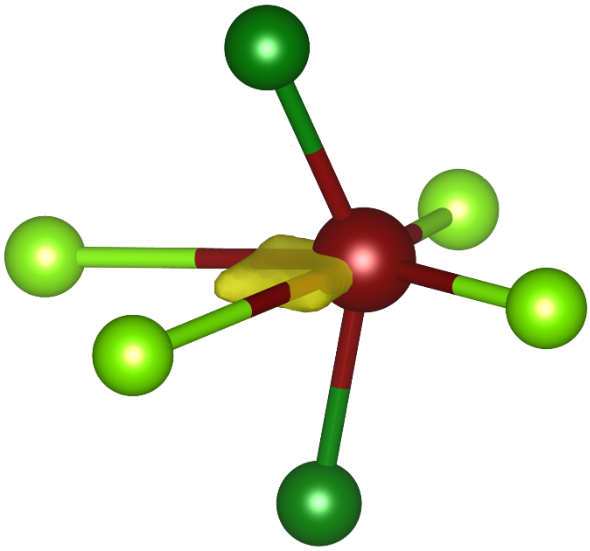

(ii)

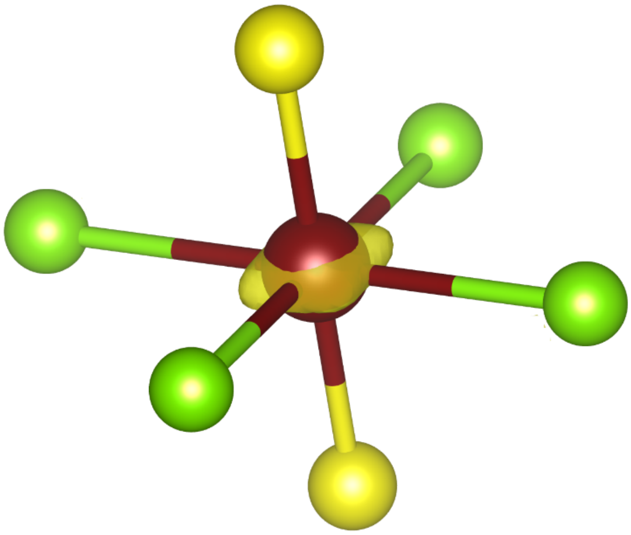

S15. Single Crystal data, data collection and structure refinement parameters of $CuBiSeCl_2$

Empirical formula	$CuBiSeCl_2$
Molecular weight (g. mol^{-1})	422.4
Temperature (K)	100
Symmetry	*Orthorhombic*
Space group	*P n m a* (62)
Unit cell dimensions (Å)	a= 8.78415(6)<br>b= 3.99803(3)<br>c= 13.13998(9)
Volume ($Å^3$)	461.467(6)
Z	4
Data Collection	
λ [Å]	0.6889
Calculated density (g cm^{-3})	6.080
Crystal shape	Platelet
Crystal dimensions (μm)	35-25-5
Color	Black
Absorption correction	Empirical
θ (min−max) (°)	2.704-34.986
μ (mm−1 ; for λ Kα = 0.56087Å)	47.29
F(000)	720
Reciprocal space recording	$-14 \leq h \leq 14$<br>$-6 \leq k \leq 6$<br>$-21 \leq l \leq 21$
No. of measured reflections	9843
No. of independent reflections	1240
I > 3σ(I) (total)	1215
Refinement	
Number of refined parameters	31
Refinement method	Sigma
Weighting scheme	
R1(F) [I > 3σ(I)]/R1(F^2) (all data, %)	0.0119 - 0.0114
wR2(F^2) [I > 3σ(I)]/wR2(F^2) (all data, %)	0.0256 - 0.0254
Goodness of Fit	1.168
Max/Min residual electronic density ($e^-/Å^3$)	0.911/-1.685

S16. Atomic Positions and Isotropic Thermal Displacement parameters for $CuBiSeCl_2$. The atomic positions listed for $CuBiSeCl_2$ are standardized.

Atom	Wyck.	*x*	*y*	*Z*	$U_{eq.}$
Bi1	4*c*	0.27145(2)	1/4	0.44748(2)	0.00532(3)
Se1	4*c*	0.26059(3)	1/4	0.80575(2)	0.00496(4)
Cu1	4*c*	0.04035(4)	1/4	0.69957(3)	0.01027(6)
Cl1	4*c*	0.05935(7)	1/4	0.10163 (4)	0.00634(9)
Cl2	4*c*	0.43403(7)	1/4	0.09546 (4)	0.00722(9)

S17. Anisotropic Thermal Parameters U_{ij} (Å^2)

Atom	U_{11}	U_{22}	U_{33}	U_{12}	U_{13}	U_{23}
Bi1	0.00565(4)	0.00600(4)	0.00430(4)	0	0.00018(2)	0
Se1	0.00491(9)	0.00628(10)	0.00369(9)	0	0.00039(7)	0
Cu1	0.00868(14)	0.01284(15)	0.00930(13)	0	0.00347(10)	0
Cl1	0.0059(2)	0.0076(2)	0.0055(2)	0	0.00011(17)	0
Cl2	0.0050(2)	0.0101(2)	0.0065(2)	0	0.00036(17)	0

S18. Main Bond Distances (Å) for $CuBiSeCl_2$

Atoms 1,2	d 1,2 (Å)	Atoms 1,2	d 1,2 (Å)
Bi1-Se1	2.74661(18)×2	Cu1-Se1	2.3852(4)
Bi1-Cl1	3.0172(6)	Cu1-Se1	2.4585(4)
Bi1-Cl2	2.6100(6)	Cu1-Cl1	2.4327(4) ×2
		Cu1-Cl2	3.4039(6)

S19. Structure refinement against powder diffraction data at room temperature

Empirical Formula	$CuBiSeCl_2$
Crystal System	Orthorhombic
Space Group	*Pnma* (62)
Cell Parameters	a = 8.8110 (7) Å<br>b = 4.0325 (6) Å<br>c = 13.106 (21) Å<br>α, β, γ = 90.0°
Cell Volume	465.686 $Å^3$
Density	6.025 g cm^{-3}
Pawley R_{wp}	2.043 %
Rietveld R_{wp}	2.36 %
R_{exp}	1.588 %
R_p	1.593 %
gof	1.490

S20 Atomic positions, isotropic displacement parameters and site occupancy factors from Rietveld refinement against powder diffraction data

Formula	Site	x	y	z	Occ.	U_{iso} ($Å^2$)
	Bi1	0.265(2)	0.25	0.4495(9)	1.0	0.00595(4)
	Se1	0.255(6)	0.25	0.809(2)	1.0	0.00456(8)
$CuBiSeCl_2$	Cu1	0.0324(6)	0.25	0.708(4)	1.0	0.0443(3)
	Cl1	0.059(1)	0.25	0.101(7)	1.0	0.0038(4)
	Cl2	0.44(1)	0.25	0.098(31)	1.0	0.0139(4)

Compositional Analysis

S21. Transmission Electron Microscopy (TEM) Images of $CuBiSeCl_2$ Particles

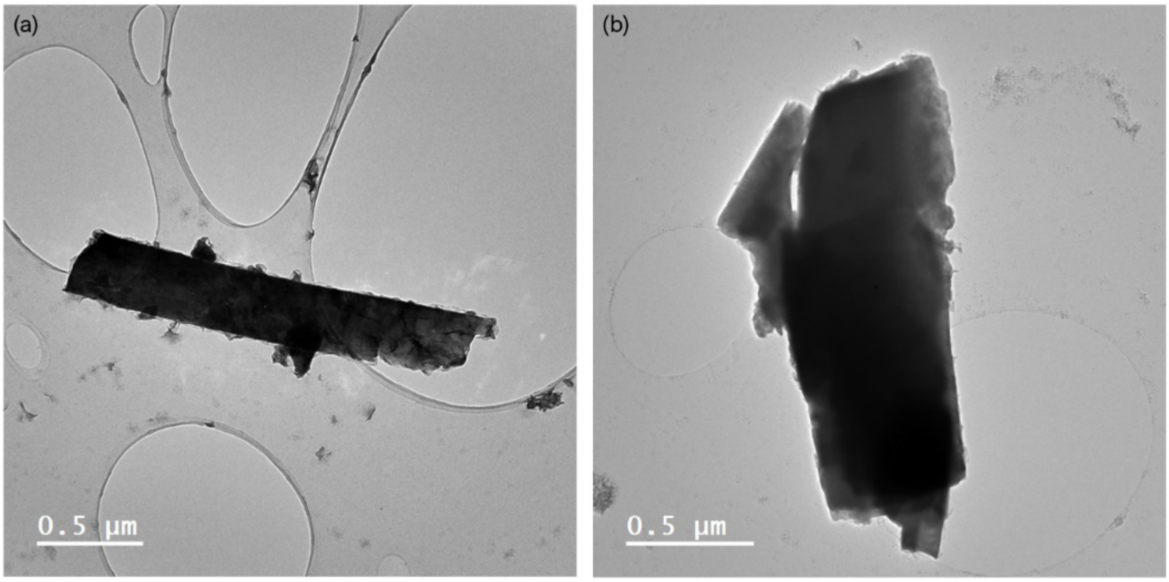

S22. Elemental Mapping of particle used for SCXRD analysis with EDX spectrum for reference

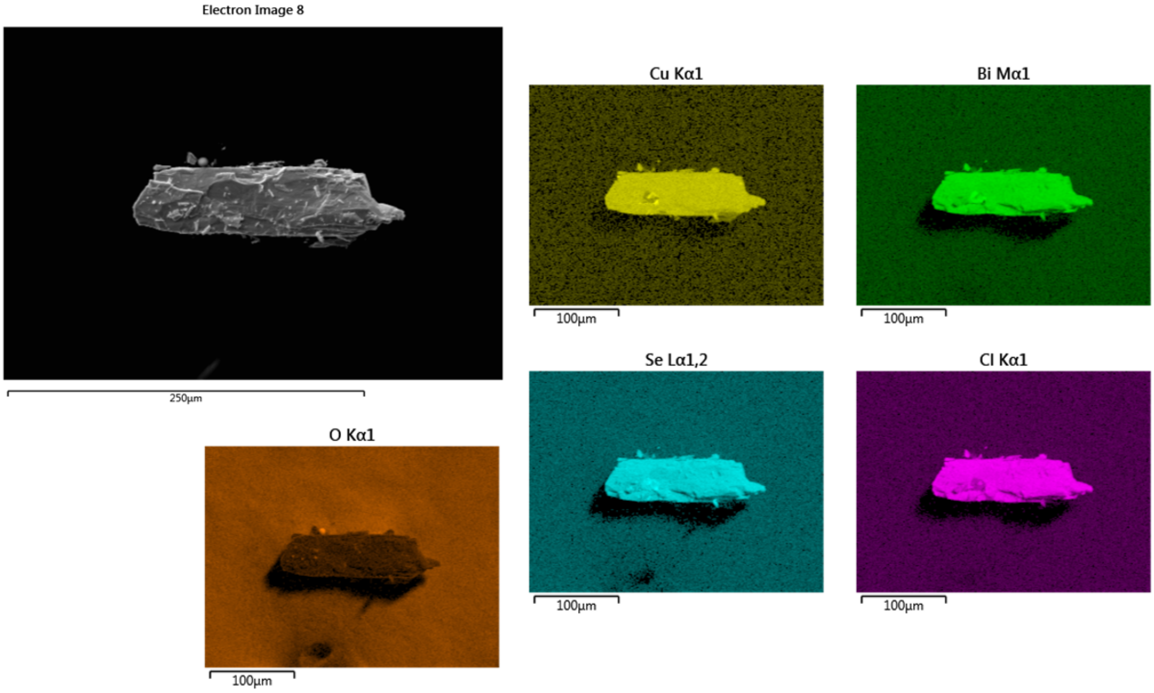

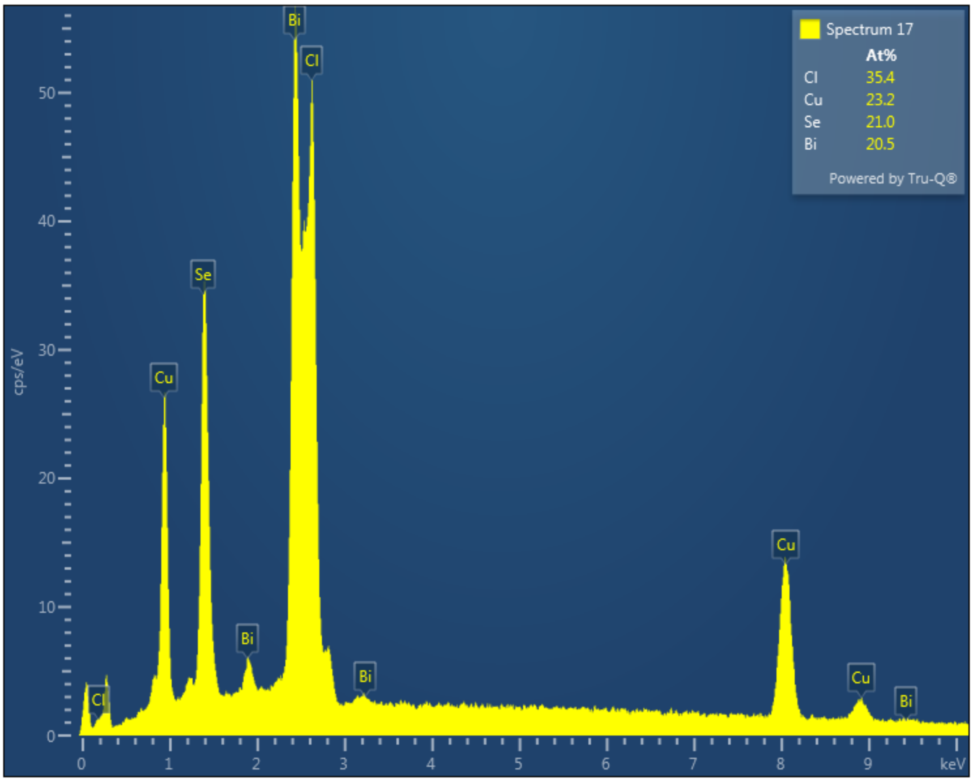

In some cases, SEM EDX is not accurate in quantitively measuring Cl in the presence of Bi due to the peak overlap, meaning that both EDX and WDX were needed to confirm the exact composition of $CuBiSeCl_2$ powder. However, with a significant Cl peak intensity as is present in the above EDX spectrum, SEM EDX elemental mapping can be used to qualitatively provide an estimate of elemental homogeneity in the material.

S23. SEM-EDX analysis of $CuBiSeCl_2$ powder pressed into pellet.

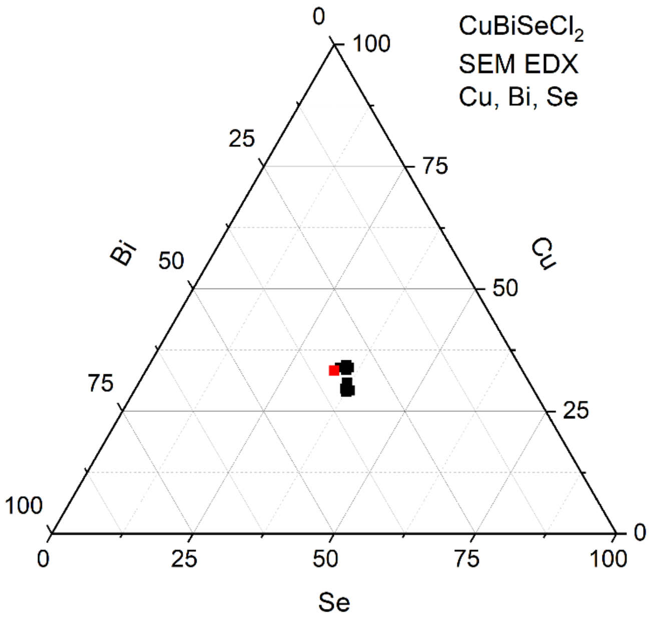

The point in red depicts the exact $Cu_{1.0}Bi_{1.0}Se_{1.0}Cl_{2.0}$ stoichiometry for reference. Some deviation from the target composition is observed in the EDX data, however the composition is confirmed through WDX analysis (S24).

S24. WDX Analysis of $CuBiSeCl_2$ powder pressed into pellet & image of pellet surface

Correction factors for WDX were determined from measurement of BiOCl and $CuCl_2$ standards

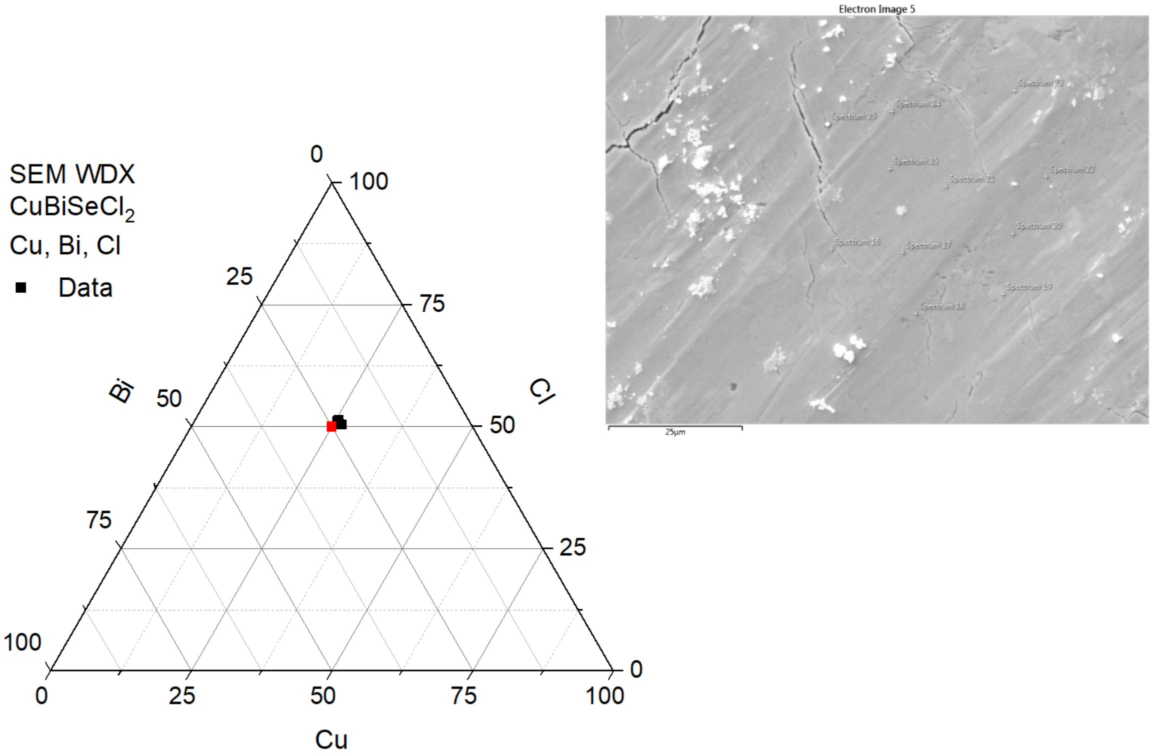

S25. Sintered $CuBiSeCl_2$ Pellet SEM Images

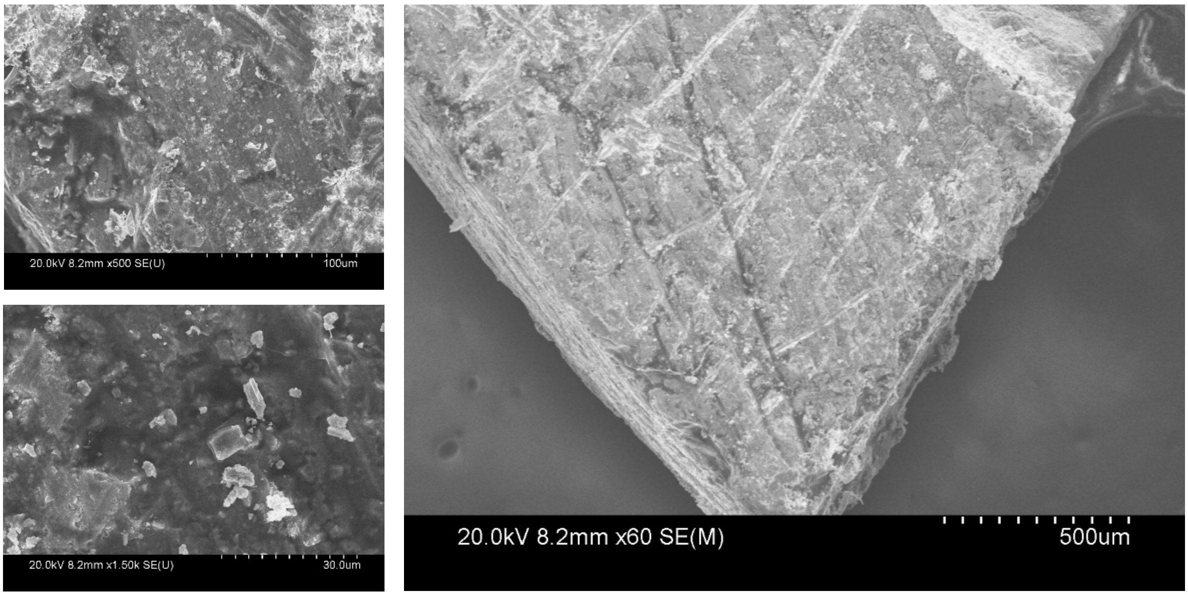

S26. SEM-EDX Analysis of <u>Sintered</u> $CuBiSeCl_2$ Pellet (88% dense)

The red dot indicates exact $CuBiSeCl_2$ stoichiometry.

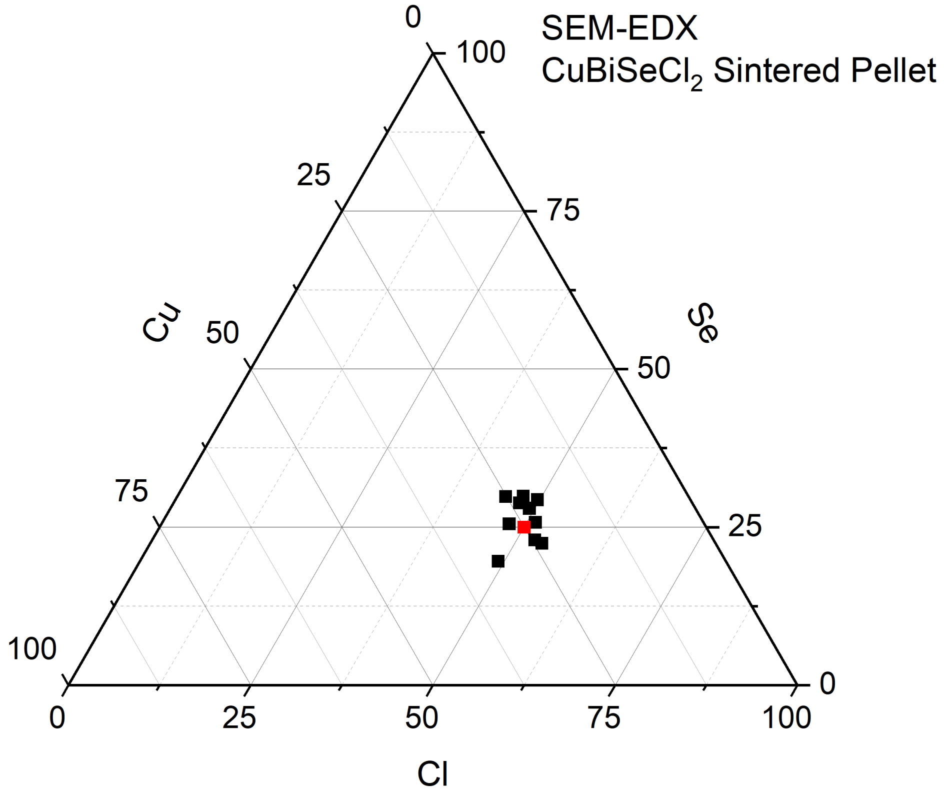

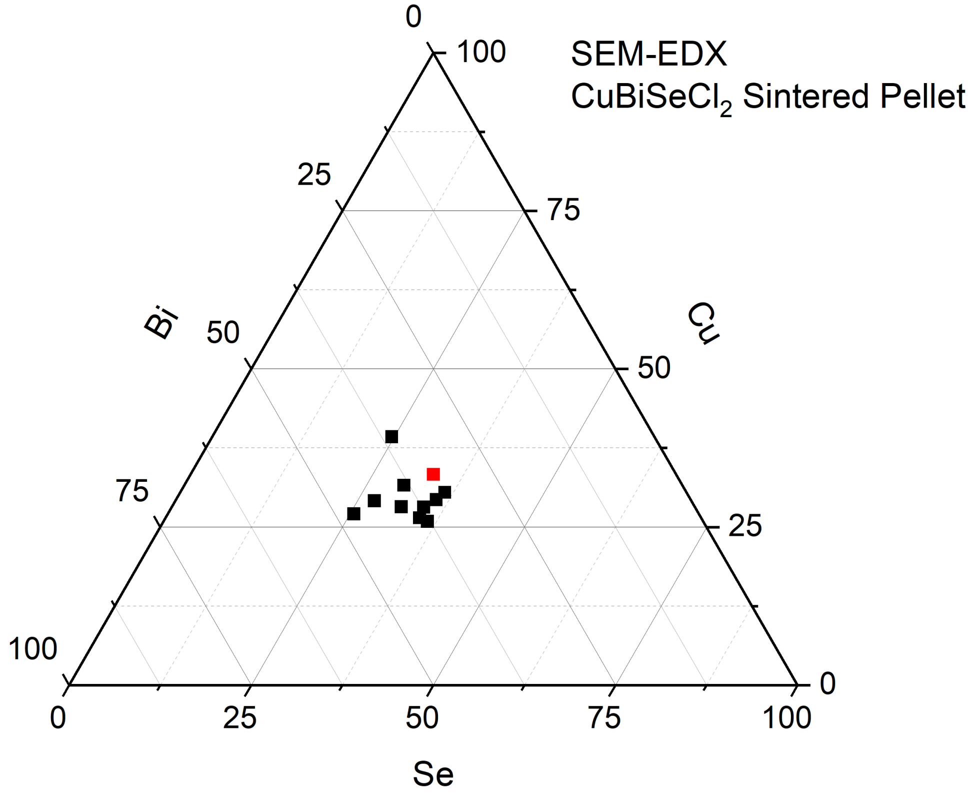

S27. PXRD data from sintered $CuBiSeCl_2$ pellet (88% dense), $CuBiSeCl_2$ powder and simulated powder pattern to highlight preferred orientation effects in the sintered pellet. Specific *[h0l]* and *[h00]* intensities are indicated to highlight the preferred orientation in these directions. The asterisk denotes the peak of which the intensity is kept constant in each PXRD dataset.

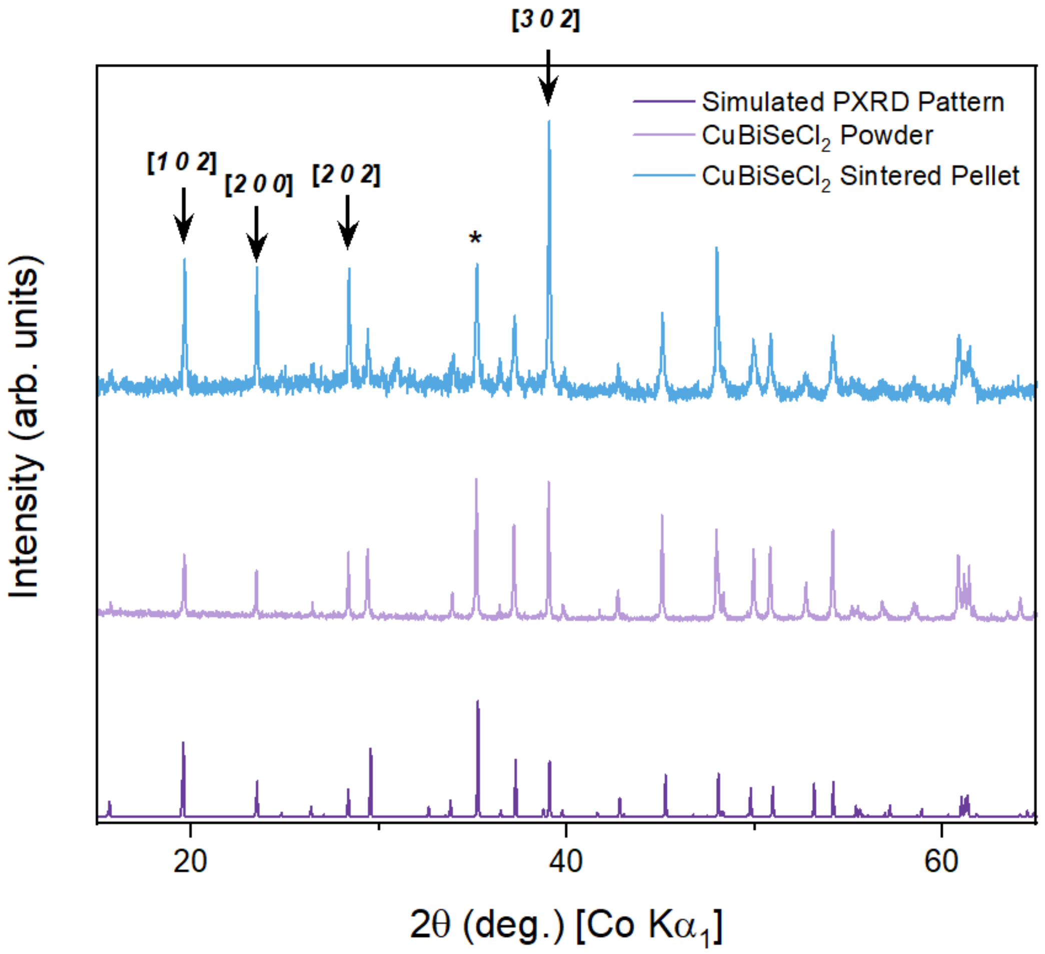

Computational Details

S28. Band structures calculated for (a) experimentally observed $CuBiSeCl_2$ (*Pnma*) (b) $CuBiSeCl_2$ in the hypothetical (*Cmcm*) structure (c) $CuBiSCl_2$ (*Cmcm*). The band structure of $CuBiSCl_2$ was calculated using the cif from [9].

(a)

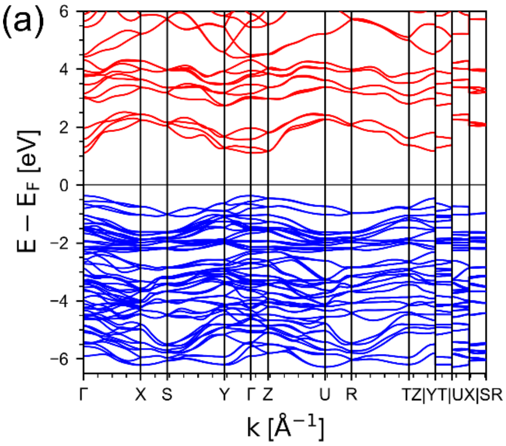

$CuBiSeCl_2$ (*Pnma*)

(b)

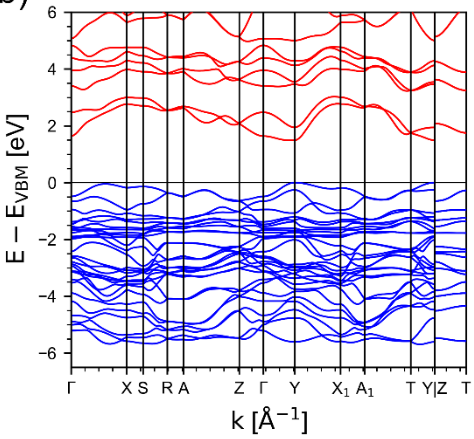

$CuBiSeCl_2$ (*Cmcm*)

(c)

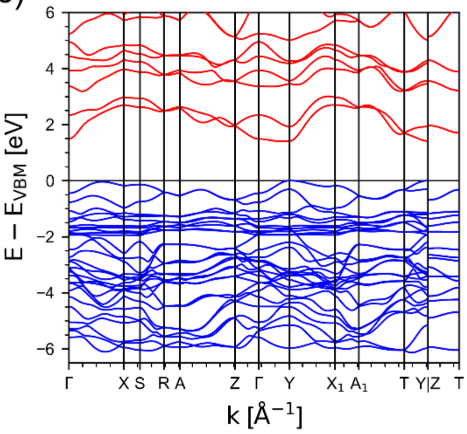

$CuBiSCl_2$ (*Cmcm*)

S29. Description of Density of States (DoS) Broadening

Density of States (DoS) calculations use Density Functional Theory (DFT) to describe the ground state valence band of a material based on its structure. X-Ray Photoemission Spectroscopy (XPS) measures the photo-electrons released from a material as a result of x-rays of a known energy irradiating the sample. DoS calculations do not account for sources of error, such as the spectrometer resolution, and realistic physical process, such as life-time broadening, that occur during experimental photoemission processes. Some deviation is always expected between experiment and calculation, since the process of photoemission perturbs electrons from the ground state. However, adjustments made to the original DoS data allow for closer comparison between XPS and DoS data by accurately representing realistic photoemission effects. Photoionisation cross sections from Scofield[17] were applied to each orbital to account for the different photoemission probabilities of each species.[18] The data were also convolved with Gaussian (width 0.25 eV) and Lorentzian functions (width 0.25 eV[19]) to account for spectrometer resolution and lifetime broadening effects, respectively. Modifying the DoS in this way allows for closer comparison between computational calculations and experimental measurement of the valence band. After these convolutions, the DoS were shifted by 1.04 eV to align with the XPS data calibrated with respect to the Fermi edge of Ag. The background was also subtracted from the experimentally determined XPS data, and the data was scaled accordingly to match the DoS. Since the DoS was calculated in terms of $E\text{-}E_{VBM}$, the DoS was inverted along the x axis to allow for comparison with the VB.

S30. As-calculated DoS for $CuBiSeCl_2$ (left) DoS after photoionisation cross section corrections and convolution with Gaussian and Lorentzian functions. (right)

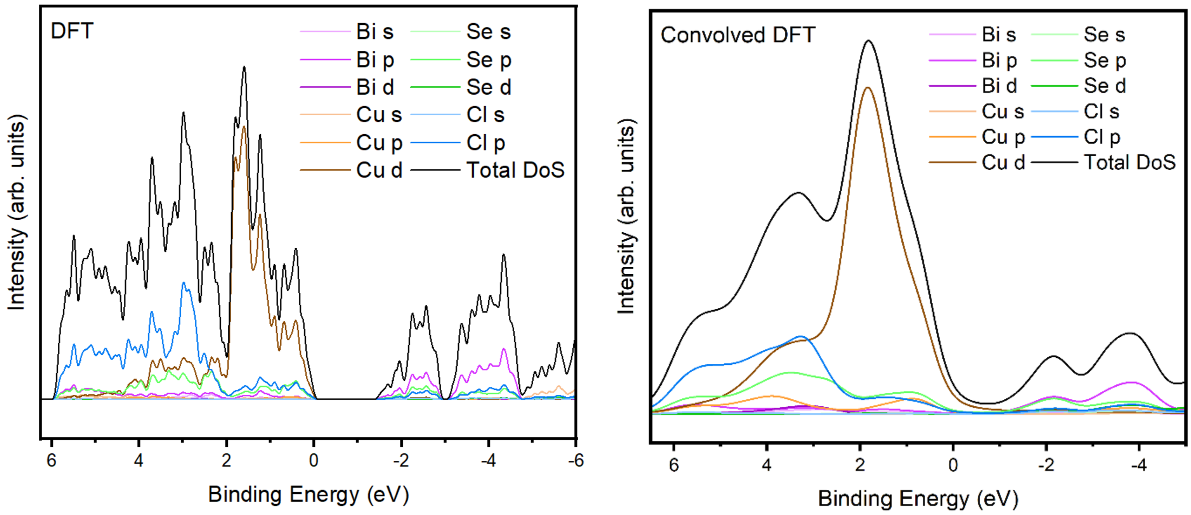

Electronic Structure

S31. XPS Survey Scan

All expected chemical species are present. Presence of the O 1s core level indicates a minor amount of surface oxidation, due to exposure to air during transit of the sample.

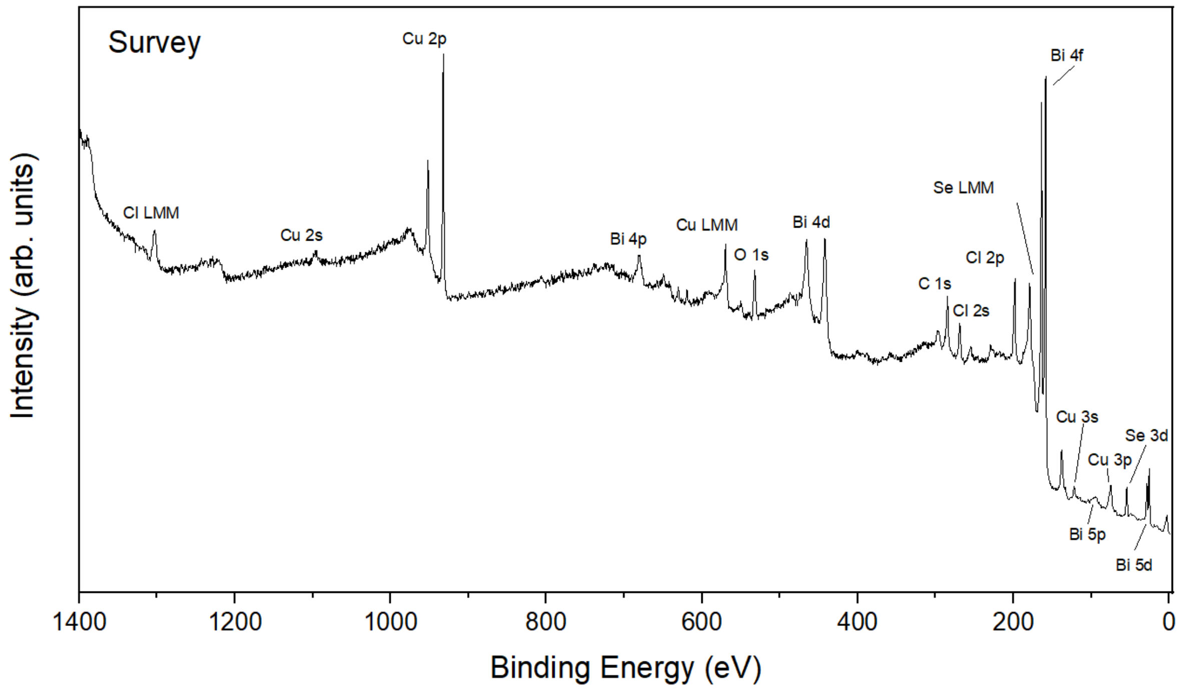

S32. Description of Band Alignment Determination

Band alignments for $CuBiSeCl_2$ were determined from measurement of the Secondary Electron Cut-off (SEC) region, shown in S33. The spectrometer work function (ϕ_s) was first determined from the SEC using Eq. 1.

$$1. \quad \phi_s = h\upsilon - E_{SEC}$$

Here, $h\upsilon$ is the photon energy and E_{SEC} is the energy of the onset of the SEC region. From this, the Ionisation Potential (IP) was calculated using Eq. 2.

$$2. \quad IP = \phi_s + E_{VBM}$$

Where E_{VBM} is the binding energy of the Valence Band Maximum (VBM). The band alignments can thus be calculated using the IP, shown in S34.

S33. Secondary Electron Cut-off (SEC) Region

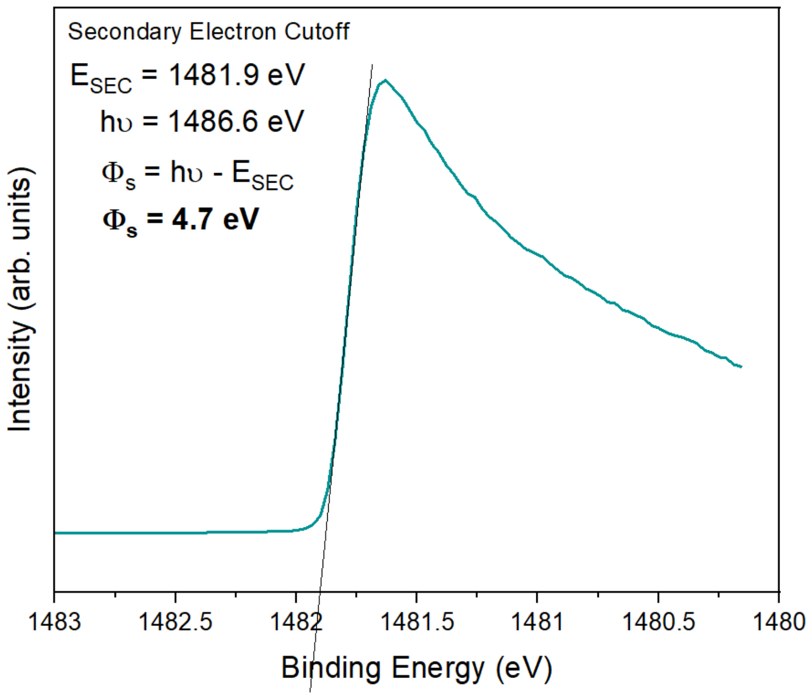

S34. Determination of Ionisation Potential using position of Valence Band Maximum (VBM)

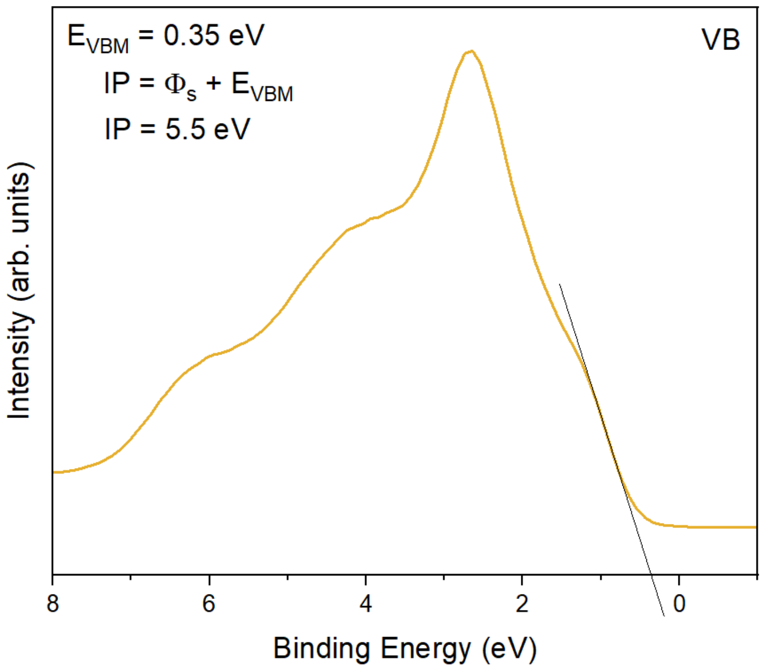

Environmental Stability

S35. - PXRDs of $CuBiSeCl_2$ powder left in ambient air for 9 weeks. The BiOCl PXRD data is simulated from [20].

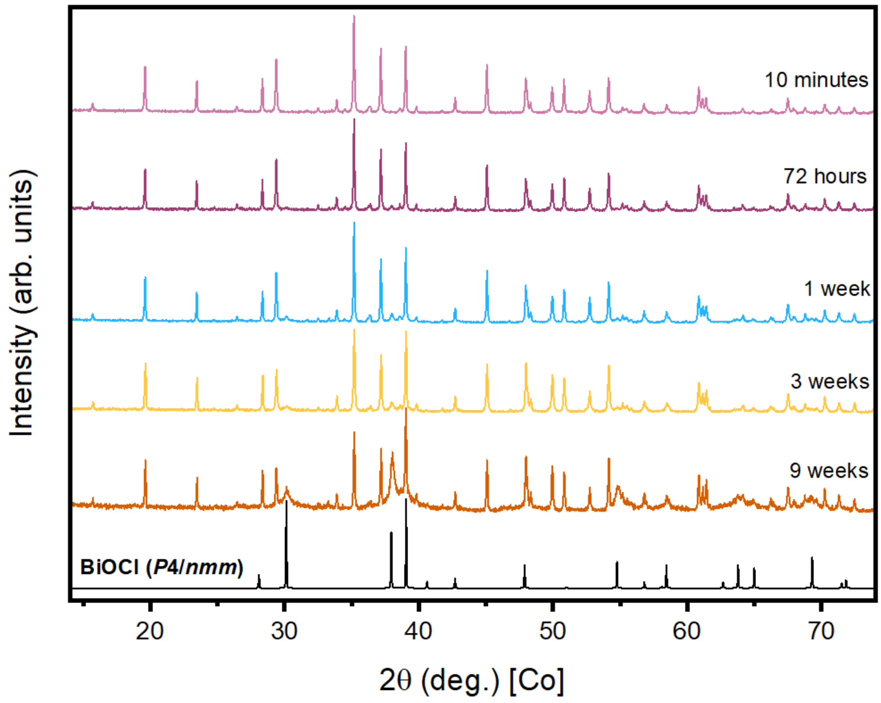

S36. - PXRD data of $CuBiSeCl_2$ powder before / after exposure to water. The BiOCl PXRD data is simulated from [20].

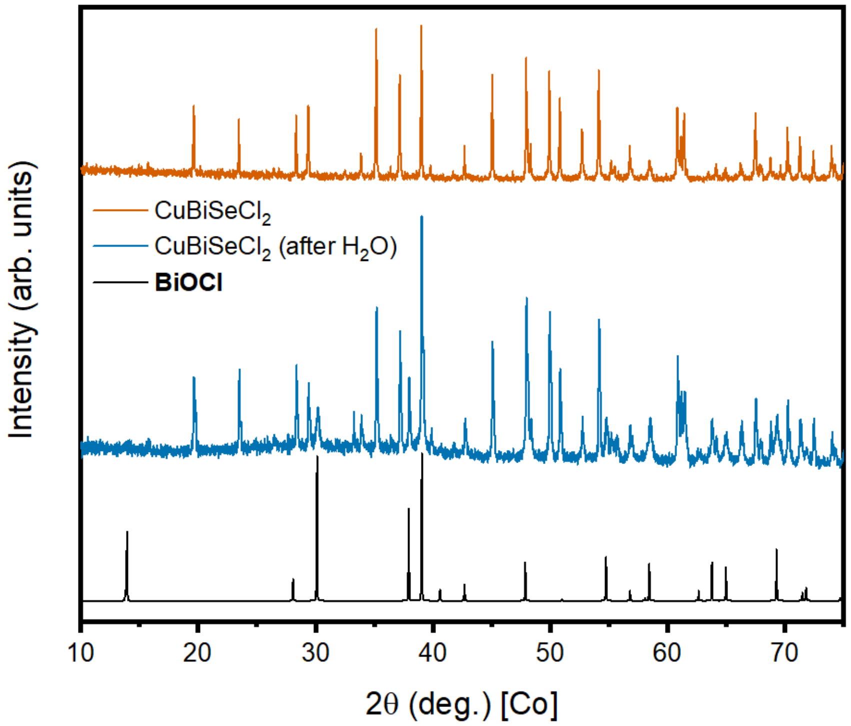

Thermal Properties

S37. Description of mathematical equations used for thermal conductivity modelling

The minimum thermal conductivity (κ_{min}) of $CuBiSeCl_2$ was estimated using the Cahill model, [18] whereby the transfer of energy is enabled by the nearest neighbour interactions involved in a random walk of coupled quantum harmonic oscillators. This is described using three Debye integrals, which are taken over three sound modes (two transverse and one longitudinal) in Eq. 3.

$$3.\ \ \kappa_{min} = \left(\frac{\pi}{6}\right)^{1/3} k_B n^{2/3} \sum_i v_i \left(\frac{T}{\Theta_i}\right)^2 \int_0^{\Theta_i/T} \frac{x^3 e^x}{(e^x-1)^2} dx$$

Where n is the number density of atoms, v_i is the speed of sound in each sound mode, and Θ_i is the cut-off frequency (Debye temperature) for each polarization:

$$\Theta_i = v_i \left(\hbar/k_B\right) (6\pi^2 n)^{1/3}$$

The diffuson-mediated thermal conductivity (κ_{diff}) was also calculated for $CuBiSeCl_2$ using the model developed by Agne et al.[19] Equation 4 approximates the limit of diffusive thermal conductivity in a material.

$$4.\ \ \kappa_{diff}(T) \approx \frac{n^{-2/3} k_B}{2\pi^3 v_s^3} \left(\frac{k_B T}{\hbar}\right)^4 \int_0^{0.95\frac{\theta_D}{T}} \frac{x^5 e^x}{(e^x-1)^2} dx$$

Where v_s is the arithmetic average speed of sound and θ_D is the Debye temperature.

S38. Description of calculation of Phonon Mean Free Path (l_{ph}), calculated using the method described in [21].

The phonon mean free paths for θ_{D1} and θ_{D2} extracted from modelling of the heat capacity data measured for $CuBiSeCl_2$ were calculated using the equation for the lattice thermal conductivity (κ_{latt}):

$$5.\quad \kappa_{latt} = \frac{1}{3} c_p l_{ph} v_s$$

Where c_p is the specific heat capacity. Here, we treat $\kappa_{tot} \approx \kappa_{latt}$ given that the electronic contribution to the lattice thermal conductivity is negligible as confirmed by resistivity measurements. This equation is rearranged to:

$$l_{ph} = \frac{3\kappa_{tot}}{c_p v_s \rho}$$

Where the material density (ρ) is included to return a phonon l_{ph} in metres.

The mean free paths determined from this method are shown in the table S39.

S39. Calculated Phonon Mean Free Paths

**Debye Temperature (θ_D) [K]**	**Velocity of Sound (v_s) [ms^{-1}]**	**Phonon Mean Free Path (l_{ph}) [m]**
**320**	3059.45	1.54
**138**	1319.39	3.56

S40. Specific Heat Modelling Prefactors

**Compound**	θ_{D1} **(K)**	Θ_{D2} **(K)**	θ_{E1} **(K)**	θ_{E2} **(K)**	**γ ($Jmol^{-1}K^{-2}$)**
**$CuBiSeCl_2$**	320	138	60	28	0.00009
Prefactors	0.618	0.28	0.10	0.002	/

As required, the Debye and Einstein modelling prefactors sum to 1.

S41. Modelling of Specific Heat Capacity Data using (i) 1 Debye term (ii) 1 Debye and 1 Einstein term (iii) 2 Debye terms and 1 Einstein term. No linear contributions were used in the fitting shown here, and all Debye and Einstein prefactors summed to 1 in each case.

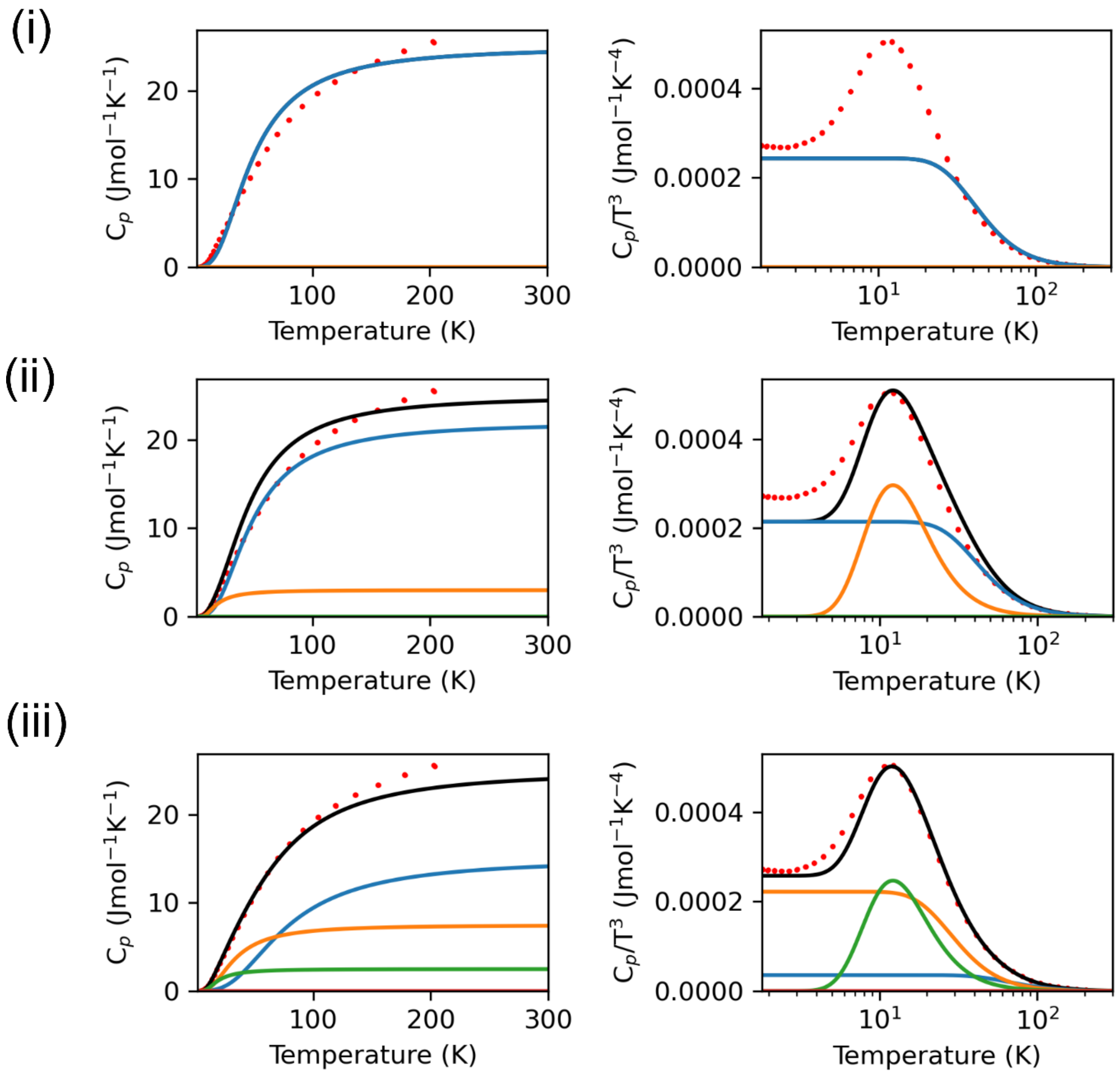

References

1. Ismail, F. M.; Hanafi, Z. M., Some Physico-Chemical Properties of Bismuth Chalcogenides x-Ray Photoelectron and Diffuse Reflectance Spectra. *Z. Phys. Chem.* **1986,** 267O, (1), 667-672.
2. Dharmadhikari, V. S.; Sainkar, S. R.; Badrinarayan, S.; Goswami, A., Characterisation of thin films of bismuth oxide by X-ray photoelectron spectroscopy. *J. Electron Spectrosc. Relat. Phenom.* **1982,** 25, (2), 181-189.
3. Mytilineou, E.; Kounavis, P.; Chao, B. S., A study of n-type conduction in amorphous chalcogenide sputtered films. *J. Phys. Condens. Matter.* **1989,** 1, (28), 4687-4695.
4. Rufus, I. B.; Ramakrishnan, V.; Viswanathan, B.; Kuriacose, J. C., Surface characterization of $CdS_{0.62}Se_{0.38}$ by X-ray photoelectron spectroscopy. *J. Mater. Sci. Lett.* **1992,** 11, (5), 252-254.
5. Sobol, P. E.; Nelson, A. J.; Schwerdtfeger, C. R.; Stickle, W. F.; Moulder, J. F., Single Crystal $CuInSe_2$ Analysis by High Resolution XPS. *Surf. Sci. Spectra* **1992,** 1, (4), 393-397.
6. Van Der Laan, G.; Westra, C.; Haas, C.; Sawatzky, G. A., Satellite structure in photoelectron and Auger spectra of copper dihalides. *Phys. Rev. B* **1981,** 23, (9), 4369-4380.
7. Sesselmann, W.; Chuang, T. J., The interaction of chlorine with copper. *Surf. Sci.* **1986,** 176, (1-2), 32-66.
8. Vasquez, R. P., CuCl by XPS. *Surf. Sci. Spectra* **1993,** 2, (2), 138-143.
9. Ruck, M.; Poudeu, P. F. P.; Söhnel, T., Synthese, Kristallstruktur und elektronische Bandstruktur der isotypen Sulfidchloride $CuBiSCl_2$ und $AgBiSCl_2$. *Z. anorg. allg. Chem.* **2004,** 630, 63-67.
10. Doussier, C.; Moëlo, Y.; Léone, P., Synthesis and crystal structures of four new bromo-chalcogenides: $MnSbS_2Br$, $MnBiSe_2Br$ and two allotropic forms of $MnSbSe_2Br$. Crystal chemistry of the $MnPnQ_2X$ family (Pn = Sb, Bi; Q = S, Se; X = Cl, Br, I). *Solid State Sci.* **2006,** 8, 652-659.
11. Bärnighausen, H., Group–subgroup relations between space groups: a useful tool in crystal chemistry. *Commun. Math. Chem* **1980,** 9, 139 - 175.
12. Müller, U., Kristallographische Gruppe-Untergruppe-Beziehungen und ihre Anwendung in der Kristallchemie. *Z. Anorg. Allg. Chem.* **2004,** 630, (11), 1519-1537.
13. Müller, U., *International Tables for Crystallography Volume A1: Symmetry relations between space groups*. John Wiley & Sons: Chichester, United Kingdom, 2010.
14. Müller, U., *Symmetriebeziehungen zwischen verwandten Kristallstrukturen*. Vieweg+Teubner Verlag: Wiesbaden, Germany, 2012.
15. Brown, I. D., Accumulated Table of Bond Valence Parameters. In IUCr: 2020.
16. Shields, G. P.; Raithby, P. R.; Allen, F. H.; Motherwell, W. D. S., The assignment and validation of metal oxidation states in the Cambridge Structural Database. *Acta Crystallogr. B: Struct. Sci.* **2000,** 56, (3), 455-465.
17. Scofield, J. H. *Theoretical photoionization cross sections from 1 to 1500 keV*; Office of Scientific and Technical Information (OSTI): 1973.
18. Kalha, C.; Fernando, N. K.; Bhatt, P.; Johansson, F. O. L.; Lindblad, A.; Rensmo, H.; Medina, L. Z.; Lindblad, R.; Siol, S.; Jeurgens, L. P. H.; Cancellieri, C.; Rossnagel, K.; Medjanik, K.; Schönhense, G.; Simon, M.; Gray, A. X.; Nemšák, S.; Lömker, P.; Schlueter, C.; Regoutz, A., Hard x-ray photoelectron spectroscopy: a snapshot of the state-of-the-art in 2020. *J. Condens. Matter Phys.* **2021,** 33, (23), 233001.
19. Whittles, T. J., Electronic Characterisation of Earth-Abundant Sulphides for Solar Photovoltaics. *Springer Theses* **2018**.
20. Bannister, F. A., The crystal-structure of the bismuth oxyhalides. *Mineral Mag* **1935,** 24, (149), 49-58.
21. Daniels, L. M.; Ling, S.; Savvin, S. N.; Pitcher, M. J.; Dyer, M. S.; Claridge, J. B.; Slater, B.; Corà, F.; Alaria, J.; Rosseinsky, M. J., A and B site doping of a phonon-glass perovskite oxide thermoelectric. *J. Mater. Chem. A.* **2018,** 6, (32), 15640-15652.
